# Single-cell melanoma transcriptomes depicting functional versatility and clinical implications of STIM1 in the tumor microenvironment

**DOI:** 10.7150/thno.54134

**Published:** 2021-03-05

**Authors:** Henry Sung-Ching Wong, Wei-Chiao Chang

**Affiliations:** 1 Department of Clinical Pharmacy, School of Pharmacy, Taipei Medical University, Taipei, Taiwan.; 2 Faculty of Pharmacy, University of Ahmad Dahlan, Yogyakarta, Indonesia.; 3 Taipei Medical University Research Center of Cancer Translational Medicine Taipei, Taiwan.; 4 Integrative Research Center for Critical Care, Wan Fang Hospital, Taipei Medical University, Taipei, Taiwan.; 5 Division of Nephrology, Department of Internal Medicine; Department of Medical Research, Shuang Ho Hospital, Taipei Medical University, New Taipei City, Taiwan.; 6 Department of Pharmacy, Wan Fang Hospital, Taipei Medical University, Taipei, Taiwan.

**Keywords:** single-cell transcriptomic analysis, stromal interaction molecule 1 (*STIM1*) melanoma tumor microenvironment, immune landscape.

## Abstract

**Rationale:** Previous studies have implicated the functions of stromal interaction molecule 1 (*STIM1*) in immunity and malignancy, however, the specificity and effects of* STIM1* expression in malignant and non-malignant cells in the tumor microenvironment are unclear.

**Methods:** In the current study, we posed two central questions: (1) does *STIM1* expression elicit different cellular programs in cell types within the melanoma tumor microenvironment (2) whether the expression of* STIM1* and* STIM1*-coexpressed genes (SCGs) serve as prognostic indicators of patient's outcomes? To answer these questions, we dissected cell-specific* STIM1*-associated cellular programs in diverse cell types within the melanoma tumor microenvironment by measuring cell-type specificity of* STIM1* expression and SCGs.

**Results:** A distinct set of SCGs was highly affected in malignant melanoma cells, but not in the other cell types, suggesting the existence of malignant-cell-specific cellular programs reflected by* STIM1* expression. In contrast to malignant cells,* STIM1* expression appeared to trigger universal and non-specific biological functions in non-malignant cell types, as exemplified by the transcriptomes of macrophages and CD4^+^ T regulatory cells. Results from bioinformatic analyses indicated that SCGs in malignant cells may alter cell-cell interactions through cytokine/chemokine signaling and/or orchestrate immune infiltration into the tumor. Moreover, a prognostic association between SCGs in CD4^+^ T regulatory cells and patient's outcomes was identified. However, we didn't find any correlation between SCGs and responsiveness of immunotherapy.

**Conclusions:** Overall, our results provide an integrated biological framework for understanding the functional and clinical consequences of cell-specific* STIM1* expression in melanoma.

## Introduction

Melanomas are considered as curable if patients are diagnosed early then the tumor can be surgically resected 1. Transformation of melanoma cells associated with somatic mutations in the proto-oncogenes, B-Raf (BRAF) and Neuroblastoma RAS (NRAS), allowing tyrosine kinase inhibitors (*e.g.*, vemurafenib, dabrafenib, and trametinib) to be successfully applied as melanoma treatments 2, 3. Nevertheless, the prognosis of melanoma patients with regional metastasis remains poor. The clinical phenotype of melanoma patients depends on the combined effects of intricate molecular interactions within and between malignant and non-malignant cells inside the tumor tissue. Moreover, large-scale genomic profiling on melanoma specimens have revealed high levels of intra- and inter-tumor heterogeneity. This heterogeneity and molecular complexity are likely to enhance cancer progression and limit the efficacies of current melanoma treatments 4, 5.

Physiological and pathological processes are dysregulated in melanoma tumors, including cell proliferation, invasion, migration, and apoptosis. These processes are tightly linked with known cellular and molecular signaling pathways in malignant cells. In melanoma and other cancers, calcium signaling plays a vital role in the regulation of tumorigenic pathways 6-12. Stromal interaction molecule 1 (*STIM1*), an endoplasmic reticular (ER) calcium sensor, involves in cancer-related pathological signaling via its interaction with Orai1, a pore-forming unit of the Ca^2+^ release-activated channel (CRAC) 11, 12. In addition to the role in calcium signaling in tumor cells,* STIM1* is an important modulator of a wide variety of cellular programs in non-tumor cells within the tumor microenvironment, such as cancer-associated fibroblasts (CAFs), endothelial cells and immune cells. For example, *STIM1*-dependent signaling in non-tumor cells has been proposed to affect the outcomes of melanoma patients 13-15, therefore, the biological roles and functional impacts of* STIM1* status in non-malignant cell types within the tumor microenvironment should be examined alongside ectopic* STIM1* expression within malignant cells. However, intra-tumor heterogeneity in bulky tumor specimens has limited the definition of *STIM1*-mediated clinical consequences additionally, the functional consequences of* STIM1* expression (*i.e.*, associated transcriptional landscape) in non-tumor cells within the melanoma tumor microenvironment (TME) remain largely unknown. Whether cell-specific* STIM1* overexpression occurs in primary and/or metastatic malignant tissues is still unclear.

*STIM1* is known to express highly in several cell types (*e.g.* fibroblasts 16) that may exist within cancer tissues 17 Recent technical and analytical advances allow us to dissect* STIM1* expression and enables a detailed examination of cell-specific cellular programs within the tumor microenvironment 5. Herein, we developed a computational pipeline to determine cell-specific transcriptional profiles that associated with* STIM1* expression in human melanoma. By leveraging single-cell melanoma expression data, we firstly identified gene profiles that are significantly co-expressed with* STIM1* in different cell types, defining module genes as those ranked as most significant for each cell type. Validation was carried out by analyzing gene expression of laser capture microdissection (LCM)-processed tissue to confirm the robustness of our pipeline. Functional enrichment analysis of the top-ranked genes from each cell type allowed us to define the cell-specific roles of* STIM1* expression in the tumor microenvironment. Finally, we investigated whether* STIM1*-coexpressed genes (SCGs) in each cell type could serve as prognostic indicators for melanoma patients. Our results indicated that SCGs have cell-type-specific effects in the tumor microenvironment. The SCGs developed from this study may serve as a prognostic indicator for melanoma.

## Methods

### Querying a single-cell expression dataset

Single-cell messenger (m)RNA-Seq (scRNA-Seq) melanoma data (GSE72056) 5 were acquired from the Gene Expression Omnibus (GEO) with expression values quantified as one-tenth of transcripts per million (TPM) and then logarithmically transformed. This dataset (comprising 19 melanoma patients) was compiled of scRNA-Seq profiles of metastatic melanoma malignant cells and other cells from the TME, including CAFs, endothelial cells, and several immune cell types (CD45^+^ cells). Information regarding cell types was directly acquired from annotations in the downloaded data, with putative normal adjacent cells being defined as those not annotated as malignant or as any other different type of cell.

### Processing of scRNA-Seq data

#### Quality control

For the downloaded data, we first removed duplicate genes and corrected the gene symbols to standard Human Gene Nomenclature Committee (HGNC) human gene symbols using the* HGNChelper* package. We next defined pooled expression (including both malignant and non-malignant cells) as E*_pooled_* = log_2_(TPM + 1), and performed gene-based filtering by excluding genes with an E*_pooled_* value of < 3. For cells annotated as T cells, we further classified them into CD4^+^ T helper (Th) cells, CD4^+^ regulatory T (Treg) cells, CD8^+^ T cells, and the remaining as “other T cells” using cell type-specific surface markers.

#### False negativity correction

To diminish the drop-off effect, we performed a false-negativity correction for expression values using a set of housekeeping genes. A list of 96 (gene symbol checked) housekeeping genes were acquired from supplement of GSE72056. The aim of performing false negativity correction was to down-weight the expression value of transcripts with less reliable measurement, and therefore taking quality of library preparation and cell integrity into account. Since the probability (for a transcript) of being detected is depended on relative abundance of the transcript and total amount of RNA, we thus utilized the housekeeping genes to construct expression curve for each cell. Since housekeeping genes were considered to be expressed constitutively across cells, and thus the only reason for undetectable housekeeping genes was technical errors. The detailed algorithm aa well as analytical steps for performing false-negativity correction has been described elsewhere 18.

#### Clustering

We performed clustering using the* t*-distributed stochastic neighbor embedding (*t*-SNE) algorithm implemented in the* Rtsne* package with the following parameters: dims = 2, pca = TRUE, pca_center = FALSE, pca_scale = FALSE, perplexity = 50, max_iter = 2500, and theta = 0.0.

### Identification of SCGs

For each cell type, we defined SCGs (*a.k.a. STIM1*-associated genes) as genes that were positively and significantly associated with the expression level of the* STIM1* gene in each particular cell type. To do this, we fitted an exponential dispersion model (EDM; *i.e.*, generalized linear regression with Tweedie distribution) as follows: (1) A dispersion parameter of 1.1; (2) The effect direction of a gene expression to the *STIM1* expression was assessed by beta (*β*)-coefficient (*β* > 0 as positively associated, while *β* < 0 as negatively associated); (3) The statistical robustness of a gene expression to the *STIM1* expression was assessed by *p*-value; and (4) In each cell type, genes with a* β*-coefficient of > 0 and an false discovery rates (FDR) of < 0.05 were considered to be SCGs. Notably, FDRs were calculated for multiple testing corrections (MTCs). For malignant cells, cells from different individuals (patients) were separated for an association analysis, and finally the results were meta-analyzed to identify malignant-derived SCGs using a fixed-effect model implemented in the* meta* package.

### Construction of* STIM1* scores

For each cell type, we calculated a* STIM1* score by the following method to minimize negative impacts caused by a wide variety of quality and complexity across cells. First, all genes were binned into 25 groups based on their pooled expression values. For each SCG, we next randomly sampled 100 genes from the same bin and calculated an average control value. Then, the expression of each SCG was centralized using the corresponding mean control value. Finally, centralized values of the top 100 SCGs were averaged to become the* STIM1* score. In contrast, for bulk data, we directly averaged the top SCGs to generate the* STIM1* score.

### Pathway enrichment analysis of SCGs

The Reactome pathway database was utilized for an over-representation analysis (ORA) as implemented in the* ReactomePA* package 19. For each cell type, the HGNC symbols of the top 100 SCGs were first converted to Entrez Gene identifiers 20 using the* clusterProfiler* package 21 before performing the ORA.* q*-values were calculated for the MTCs, and Reactome pathways with a* q*-value of < 0.05 were considered to be significantly enriched.

### Additional melanoma expression datasets for validation and downstream analyses

We used the* GEOquery* package to query normalized human melanoma datasets (microarrays), which included GSE3189 22, GSE65041 23, GSE78220 24, GSE4587 25 and GSE4570 26. Logarithmic transformation was applied to the downloaded expression values that had not previously been log-transformed. Moreover, two melanoma datasets (GSE7929 27, GSE1845 28) with cell line data were further downloaded. For each dataset, we performed gene-based mean-centering of expression levels for the downstream analysis. Gene expression values were mean-aggregated from probes, and further corrected for standard HGNC human gene symbols. Additionally, 471 skin cutaneous melanoma expression profiles from The Cancer Genome Atlas (TCGA) database and 974 normal skin tissue expression profiles from The Genotype-Tissue Expression (GTEx) project were acquired from the recount2 website 29. Downloaded count data were scaled by considering the samples' total coverage. After gene-based summarization by taking an average across the same genes, we used log_2_(E*_i_*+ 1) to represent the expression value of each gene* i*. Expression values were further adjusted for latent covariates using the* sva* package and corrected for standard HGNC human gene symbols. In addition, 4 melanoma datasets (including GSE22155 30, GSE65904 31, 32, GSE19234 33 and GSE53118 34, 35) with available gene expression and clinical prognostic outcome (survival endpoints: overall survival (OS) or disease specific survival (DSS)) were downloaded for survival analysis using Cox regression test.

### Transcriptome-wide gene-association and pathway-enrichment tests for malignant-derived* STIM1* scores in TCGA melanoma data

We conducted association tests to identify genes that were significantly associated with malignant-derived* STIM1* scores. Since the malignant-derived* STIM1* scores were calculated from expression values of the top 100 SCGs, we adopted the following rigorous* jackstraw* 36 permutation approach to prevent inflation of the statistical significance with several modifications: (i) for each resampling iteration, we permuted all genes; and (ii) we used* Z*-transformed malignant-derived* STIM1* scores to calculate the null-association statistics. For each gene, the significance of the association was determined by the* p* value calculated from the above method, and the direction of the association was determined using Pearson's product-moment correlation between gene expression values and malignant-derived* STIM1* scores across samples. Genes with a positive Pearson's product-moment correlation coefficient and Bonferroni-adjusted* p* value of < 0.05 were then subjected to the Reactome pathway ORA.

### Immune metagene analysis

To identify genes that were specifically expressed in diverse cell types in the TME, we utilized non-malignant single-cell profiles from GSE72056 (including CAFs, endothelial cells, and immune cells). Immune cells were further classified into B cells, T cells, natural killer (NK) cells, and macrophages based on annotations from downloaded data. We further distinguished T cells into CD8^+^ T cells (with average expressions of* CD8A* and* CD8B* of > 3.0), CD4^+^ Treg cells (with average expressions of* FOXP3* and* CD25* of > 3.5) and CD4^+^ Th cells (with an average expression of* CD4* of > 3.0 as well as expressions of* FOXP3* and* CD25* of < 2.5) using well-established cell type markers. We next identified genes that were specifically expressed in diverse cell types per three criteria as described elsewhere 37. In addition, we also adopted an immune-related cell type-specific marker gene list from a study by Bindea* et al.*^38^. For those non-malignant cell type-specific marker genes, we then averaged expression values of these genes to construct immune metagenes.

### Statistical analyses

All statistical association tests and enrichment analyses that were conducted in this study were performed using R (http://www.r-project.org/ and http://cran.r-project.org/) or Bioconductor (http://www.bioconductor.org/).

## Results

### Single-cell resolution of* STIM1* expression in human metastatic melanoma tumors

**Figure 1:**
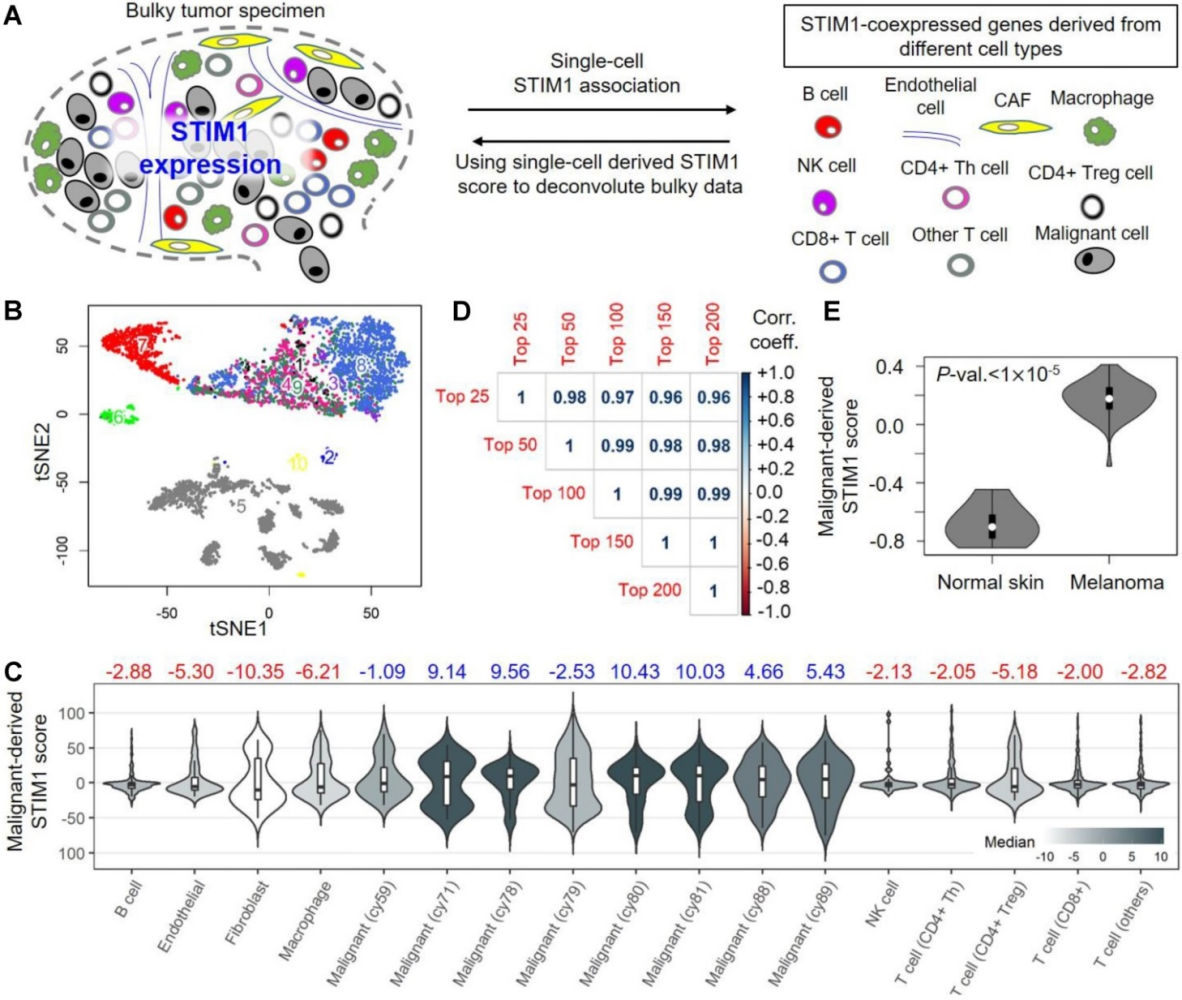
** Profiling of* STIM1* expression and its co-expressed genes in melanoma expression profiles.** (**A**) Schematic depicting our study design. Single-cell malignant melanoma data were utilized to identify* STIM1*-coexpressed genes (SCGs) in diverse cell types. The top 100 SCGs for each cell type were then adopted to construct* STIM1* scores in bulk melanoma data. (**B**) The *t*-Distributed stochastic neighbor embedding (*t*-SNE) clustering of single-cell transcriptomic profiles from 19 metastatic melanoma patients. The numbers (and colors) indicating different cell types, including 1) CD4^+^ Treg cell; 2) Endothelial cell; 3) NK cell; 4) CD4^+^ Th cell; 5) Malignant cell; 6) Macrophage; 7) B cell; 8) CD8^+^ T cell; and 9) Other T cell. (**C**) Distributions of malignant-derived* STIM1* scores in diverse cell types including malignant cells, cancer-associated fibroblasts (CAFs), endothelial cells, and several types of immune cells (GSE72056; single-cell transcriptomic data). Median values of malignant-derived* STIM1* scores are shown, with blue indicating malignant cells and red indicating non-malignant cell types. (**D**) Pearson's product-moment correlation coefficients of malignant-derived* STIM1* scores calculated using the top 25, 50, 100, 150, and SCGs derived from malignant single-cell profiles. (**E**) Malignant-derived* STIM1* scores from bulk malignant melanoma tissues were significantly higher than those from normal skin tissues (GSE3189; microarray data;* p* value < 10^-5^).

Single-cell transcriptomic data provides a better resolution across cell types Here we captured gene set reflected by *STIM1* in a cell specific profiling (Figure 1A). Malignant cells and non-malignant cells within the tumor microenvironment, including endothelial cells, CAFs and several immune cell types (B cells, macrophages, NK cells and T cells) (Supplementary Figure S1) were analyzed. Based on the T cell expression profiles, we further categorized the populations into subtypes: CD4^+^ T-helper cells (CD4Th), CD4^+^ T regulatory cells (CD4Treg), CD8^+^ T cells (CD8T), and other T cells (Supplementary Figure S2).

In the analysis of bulky RNA-seq, frequent false-negatives in single-cell RNA-seq experiments may adversely impact downstream analyses. Thus, a correction to expression profiles is performed to diminish the dropout effect by down-weighting genes that have a higher probability of being false-negatives 18. We then conducted a* t*-distributed stochastic neighbor embedding (*t*-SNE) analysis to cluster cells based on weighted expression. For malignant cells, a remarkable pattern of inter-tumor heterogeneity was observed (Figure 1B); whereas non-malignant cells tended to cluster together regardless of their cellular identity (Supplementary Figure S3). Accordingly, we analyzed malignant cells separately (by sample) and grouped non-malignant cells by the cellular identities. Based on the corrected expression profiles, the distributions of* STIM1* expression in different cell types (Supplementary Figure S4) reveal a consistent pattern of low* STIM1* expression across multiple cell types. For malignant cells, CD4^+^ Treg and fibroblasts, less than 50% cells with *STIM1* gene expression of > 0; while for the remaining cell types, less than 25% cells with *STIM1* gene expression of > 0.

We postulated that the effects of S*TIM1* may coordinate with other genes. The interrelated profiles triggered by *STIM1* may differ across cell types. We employed a stepwise bottom-up approach to: 1) identify* STIM1*-coexpressed genes (SCGs; *a.k.a. STIM1*-associated genes) in different cell types, including malignant cells in the tumor and non-malignant cells from the tumor microenvironment; 2) characterize the functional impact of SCGs in specific cell types; and 3) determine the prognostic value of SCGs derived from different cell types.

### Cell-specific identification of* STIM1* co-expression profiles in melanoma tumors

Instead of analyzing cell-specific* STIM1* expression, we integrated SCGs profiles to overcome limitations of dropout in single-cell expression profiles. Co-expression was defined as a positive and significant association with* STIM1* expression in a particular cell type. We firstly delineated transcriptome-wide associations with* STIM1* expression using an exponential dispersion model (EDM). For malignant cells, we fitted the same model in each sample and then performed a meta-analysis to pool the statistics and acquire malignant-cell-derived SCGs. Regarding to the remaining cell types, SCGs were derived independently for each cell type. Subsequently, we selected the top 100 SCGs from each cell type to construct a “*STIM1* score” (Supplementary Table S1), which could be considered as a robust estimate of* STIM1* expression from single-cell transcriptomic profiles. Then this score was applied to replace* STIM1* gene expression in the following analyses.

To examine whether the SCGs are cell-type specific, we focused on the top 100 malignant-cell-derived SCGs. The profile was used to calculate malignant-cell-derived* STIM1* scores in different cell types (Figure 1C). In malignant cells, we found significant differences between the malignant-cell-derived* STIM1* scores across individuals. Most patients expressed high values (median > 0; 6 of 8 individuals), and the remaining patients exhibited low expression values (median < 0; 2 of 8 individuals), suggesting a high level of variation in SCGs across melanomas. Moreover, we observed relatively low malignant-cell-derived* STIM1* scores in non-malignant cells (all median < 0), suggesting higher expression levels of malignant-cell-derived SCGs in malignant cells (Kruskal-Wallis rank-sum test* p*-value = 0.0041). However, a considerable subpopulation with higher *STIM1* score in CAFs, macrophages and CD4^+^ Tregs was observed (25% ~ 33.6% of cells with high *STIM1* score; Supplementary Figure S5). Previous study suggested the roles of *STIM1* in normal melanocytes, including cell proliferation, melanogenesis and pigmentation 39. Therefore, levels of malignant-derived *STIM1* score in melanoma, nevi (benign) and melanocytes (normal) were compared by using several melanoma expression datasets (including GSE4587, GSE4570 and GSE3189; Supplementary Figure S6). However, we didn't observe significant difference in malignant-cell-derived *STIM1* score level between melanomas and normal melanocytes (Mann-Whitney test *p*-values > 0.05; from GSE4587 and GSE4570). In addition, malignant-cell-derived *STIM1* score level was significantly different across melanomas and benign nevi tissue (Mann-Whitney test *p*-values < 0.05; from GSE4587 and GSE3189).

Pairwise comparisons between cell types of the same patients revealed a higher proportion of inter-class significance than intra-class significance (Supplementary Figure S7), suggesting a relatively homogeneous distribution of malignant-cell-derived* STIM1* scores from malignant or non-malignant cells. Notably, these results not only showed that malignant cells possess higher malignant-cell-derived* STIM1* scores than non-malignant cells, but the specificity of malignant-cell-derived* STIM1* scores were validated in malignant cells. Furthermore, the identification of SCGs in each cell type was accomplished by fitting the EDM with a power parameter of 1.1. We selected this power parameter based on the distribution pattern of* STIM1* expression in single-cell profiles (Supplementary Figure S4). To examine whether the results are consistent for different power parameter thresholds, we repeated the analysis using only malignant cells and various power parameters thresholds of 1.0, 1.2, 1.5 and 1.9 (Supplementary Figure S8). The associations were restricted to each patient and then samples were meta-analyzed across for the final statistical comparison. We observed a high consistency between the EDM results by different power parameters (Pearson's* r* of* Z* statistics ranged from 0.9361 to 0.9994; all* p*-values < 0.001), suggesting the robustness of statistical tests for SCGs identification.

In addition, the reliability of our analysis may depend on the number of top SCGs (*i.e.* 100 SCGs in this study) used for* STIM1* score construction. We thus applied the data from malignant cells and re-computed malignant-cell-derived* STIM1* scores using the top 25, 50, 150 and 200 SCGs. A strikingly high correlation between malignant-cell-derived* STIM1* scores derived from different numbers of genes were observed (Pearson's* r*: 0.96 ~ 1; Figure 1D). After validating the robustness of scoring paradigm, we sought to biologically validate the co-expression of genes using single-cell transcriptomic profiles. Using a group of prominent standard reference genes (*AXL*- and* MITF*-program genes that are negatively correlated with each other) to exhibit pairwise relationships, we confirmed the performance of our pipeline. As shown in Table 1,* MITF* was significantly co-expressed with predefined “*MITF*-program genes” including* TYR*,* PMEL*,* PLP1*, and* GPR143*, while a negative correlation was clearly seen between* MITF* expression and “*AXL*-program genes”, including* ANGPTL4* and* FSTL3*. Overall, these results substantiated the robustness of our pipeline for the* STIM1* score construction.

### Apply malignant-cell-derived SCGs for the deconvolution of bulky melanoma profiles

In bulky malignant melanoma tissues, the expression of* STIM1* in malignant cells is likely to be masked by other cell types (*e.g.*, immune cells). Thus, how to systematically characterize the cellular functions associated with* STIM1* expression in malignant melanoma cells remains challenging. Due to the established gene profiles that reflect the specificity (Figure 1C and Supplementary Figure S7), we hypothesized that malignant-cell-derived* STIM1* score may capture more information to reflect the* STIM1*-associated cellular programs in bulky tumors. The hypothesis was assessed by following aspects: (1) First, a published dataset from laser captured micro-dissection (LCM)-processed melanoma tumors (only included malignant cells) was analyzed (GSE65041). We considered these data to be a malignant-cell-only profile and created a ranked list on the basis of the Pearson's product-moment correlations of all genes associated with* STIM1* expression. The enrichment of the top SCGs derived from the single-cell malignant profiles in the ranked gene list was examined. As expected, a significant enrichment in the first 20-40% observations (Table 2; all minimum hypergeometric (mHG) test* p*-values < 0.05) with using the top 25, 50, 150 and 200 SCGs yielded higher degrees of enrichment was observed. (2) We asked whether the trend could be observed in data from non-malignant cells. Comparing of normal skin tissues acquired from Genotype-Tissue Expression (GTEx; 974 specimens) revealed the loss of significance of enrichment after including higher numbers of top SCGs (Supplementary Figure S9). (3) To identify whether constructed *STIM1* score is affected by infiltrating immune cells, we utilized 471 bulk melanoma data (103 primary and 368 metastatic samples) from The Cancer Genome Atlas (TCGA) project. *STIM1* score was calculated by averaging the expression values of the top 100 malignant-derived SCGs. A low correlation between malignant-derived *STIM1* score and 4 tumor purity indices 40 (ESTIMATE [Estimation of STromal and Immune cells in MAlignant Tumours using Expression data], LUMP [leukocytes unmethylation for purity], IHC [immunohistochemistry] and CPE [consensus measurement of purity estimations]) were observed (Pearson's *r*: -0.05 ~ +0.12; Supplementary Figure S10).

To directly compare the malignant-cell-derived* STIM1* scores from malignant tissue and normal counterparts, we next interrogated an expression dataset that comprised 45 bulky primary melanoma tumors and 7 normal skin lesions (GSE3189). The malignant-cell-derived* STIM1* scores for the bulky tumor data were computed by simply taking an average across the top 100 SCGs derived from the malignant single-cell data. The malignant-cell-derived* STIM1* scores of melanoma tissues were significantly higher than those for normal skin tissues (approximative Fisher-Pitman permutation* p*-value < 0.00001; Figure 1E), which was in line with previous evidence that melanoma biopsies exhibit higher* STIM1* expression than control biopsies 12. Together, our observations confirmed that the malignant-cell-derived* STIM1* scores obtained from single-cell malignant profiles achieved great results in capturing information about malignant-cell-specific SCGs in bulk expression data.

**Figure 2:**
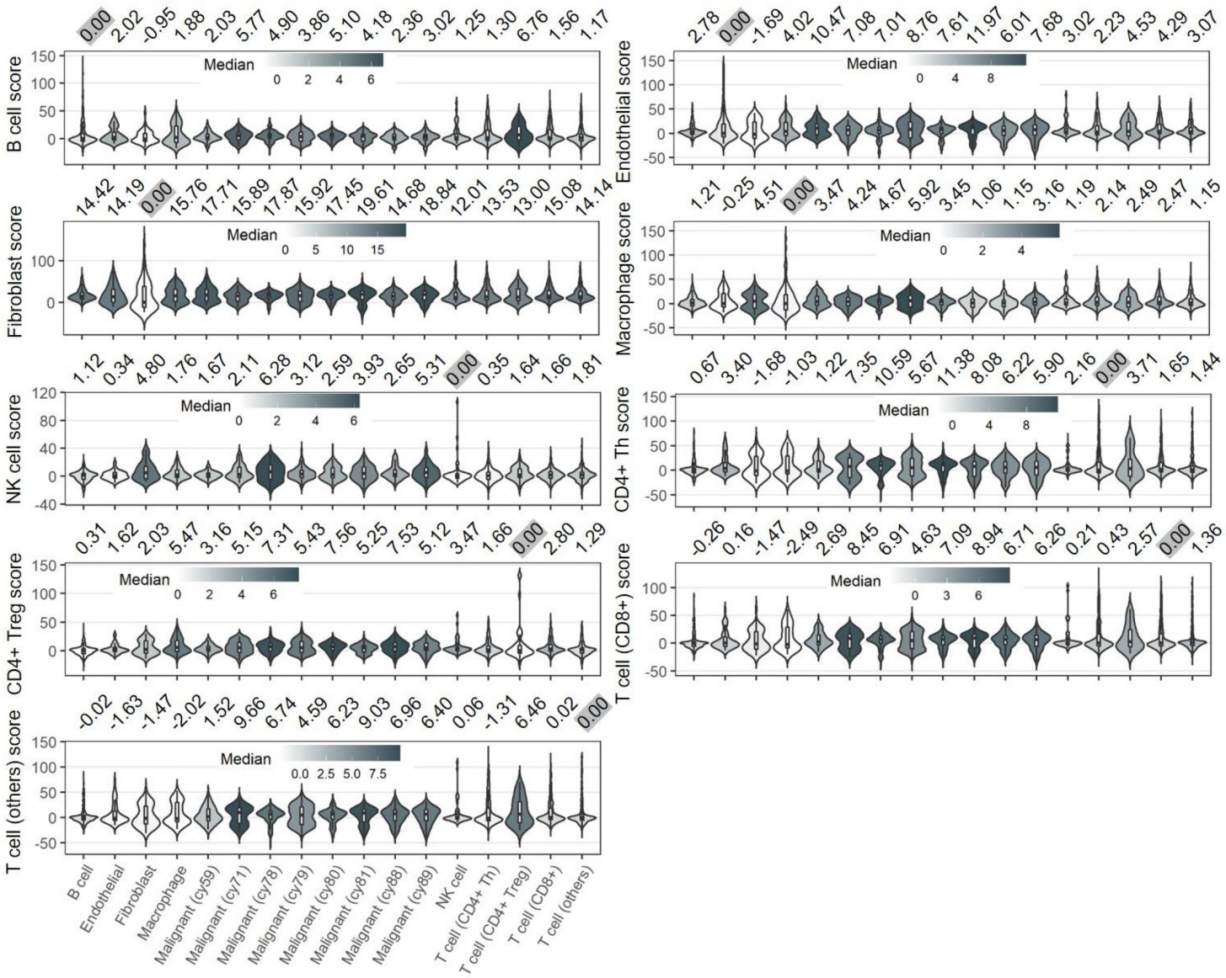
**Distributions of non-malignant cell types-derived *STIM1* scores in 9 cell types.** The numbers on the top of each panel indicate median value of corresponding cell-type derived *STIM1* score. The *STIM1* scores were median-scaled by the cell type (scaled to 0 and highlighted in grey color) used for score construction. In contrast to malignant-derived *STIM1* score (**Figure 1C**), non-malignant cells-derived *STIM1* score showed less specificity in the cell type that it derived from, suggesting the pervasive expression of non-malignant cell types' SCGs in another cell types.

We next examined the specificity of SCGs calculated from non-malignant cell types. However, the* STIM1* scores derived from non-malignant cell types were not specific when compared the difference among the non-malignant cell types (Figure 2 and Supplementary Figure S11). For example, across non-malignant cells, the expression of* STIM1* is co-regulated with similar group of SCGs which may share pathophysiological pathways. Therefore, we may not simply deduce the specificity property of SCGs (and* STIM1* score) that derived from non-malignant cells, considering the ubiquitous expression of these genes in non-malignant (even in malignant) cell types.

### Functional enrichment analysis implicates calcium elevation and immune-related pathways in* STIM1* expressing malignant cells

**Figure 3:**
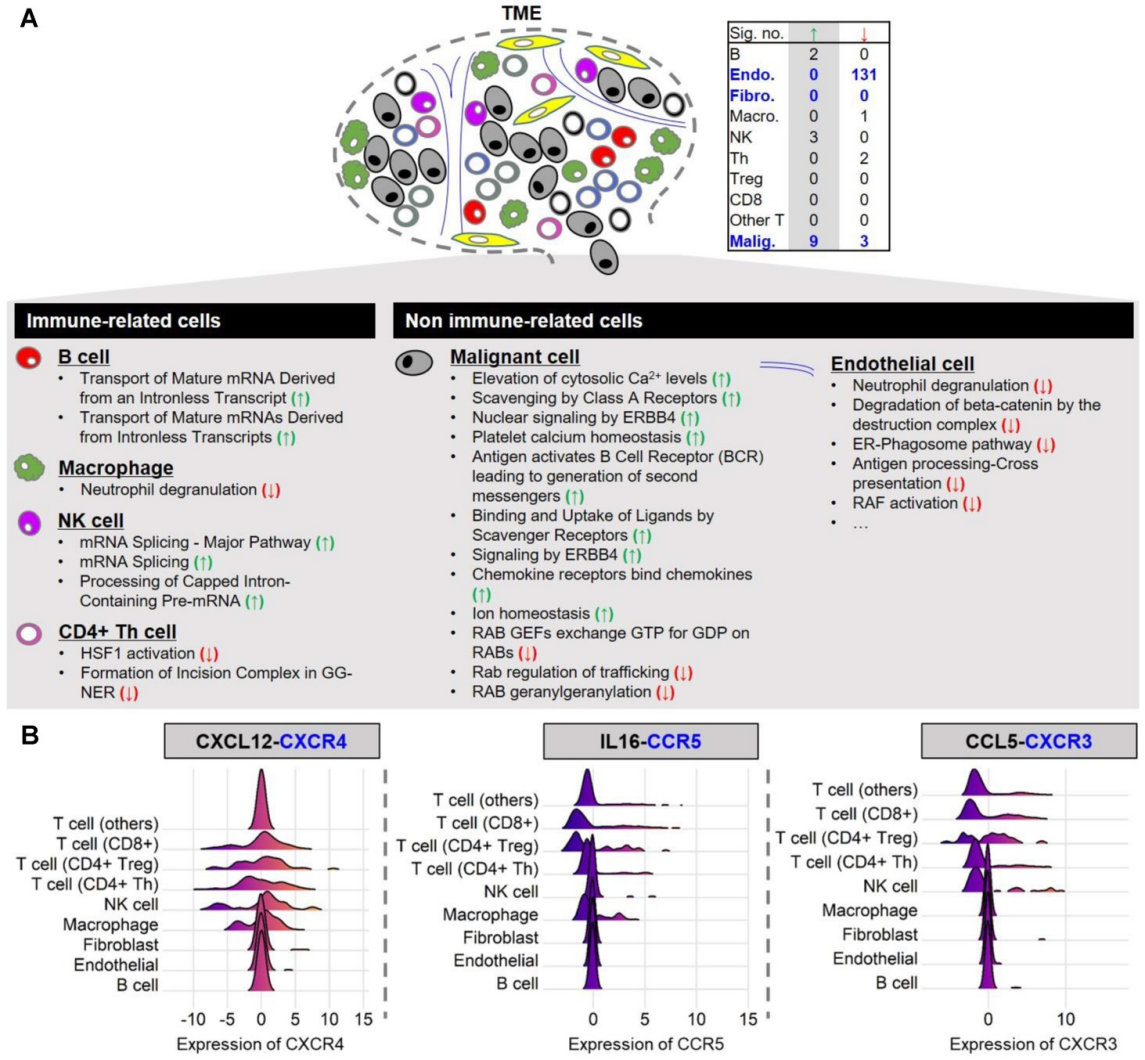
** Reactome pathway enrichment analysis of the top 100 SCGs and the distributions of expression levels of receptor genes in diverse non-malignant cell types.** (**A**) Table showing the number of significant over-represented (green arrow) and under-represented (red arrow) genes among the top 100 SCGs in Reactome pathways for different cell types (over-representation analysis (ORA);* q*-value < 0.05). Significant over-represented (green arrow) and under-represented (red arrow) Reactome pathways for each cell type are listed. (**B**) Only the distributions of receptor genes (but not ligand genes) are illustrated using single-cell malignant melanoma transcriptomes (GSE72056). In the header, receptor genes (left:* CXCR4*; middle:* CCR5*; right:* CCR3*) are colored blue, while corresponding ligand genes (co-expressed with the* STIM1* gene in malignant melanoma cells; left:* CXCL12*; middle:* IL16*; right:* CCL5*) are colored black.

To evaluate the functional similarity of SCGs among different cell types, we conducted an over-representation analysis (ORA) on single-cell data using the Reactome Pathway Database (Figure 3A). We first performed enrichment tests for the top 100 malignant-cell-derived SCGs. A significant enrichment (*q*-value < 0.05) for pathways related to Ca^2+^ signaling was found including elevation of cytosolic calcium levels, platelet calcium homeostasis, and ion homeostasis. The identified SCGs are consistent with the known functions of* STIM1* in calcium signaling. Since functionally enriched genes were associated with the expression level of* STIM1* in malignant melanoma cells, these results imply* STIM1* expression is positively associated with particular calcium-mediated biological functions, including cell cycle regulation and cell migration 11, 12. Furthermore, we also observed enrichment of malignant-cell-derived SGCs for immune-related pathways, such as chemokine signaling, implying that malignant cells may stimulate immune cell responses via* STIM1*-mediated calcium signaling. Subsequently, we probed the enrichment of SCGs from non-malignant cell types in melanoma. Strikingly, only a few over-represented pathways were identified. Taken together, these results indicated that* STIM1* expression is associated with a unique cellular program in malignant cells, in contrast to its role in non-malignant cell types.

To validate the functions of malignant-cell-derived SCGs in a larger sample size, an independent dataset from TCGA were subjected for pathway enrichment analysis. To do so, we constructed a malignant-cell-derived* STIM1* score from 277 melanoma specimens with tumor purity (IHC) > 80% (44 primary and 233 metastatic) by averaging the top 100 malignant-cell-derived* STIM1*-coexpressed profiles. According to previous results, these genes were highly expressed in malignant cells but not in another cell types. We, thus, postulated that the malignant-derived* STIM1* score (*i.e.* average value in bulky tissues) can be utilized as a surrogate for malignant-cell-specific functional activation of* STIM1* expression in bulky cancer specimens (Supplementary Figure S12). Here, a total of 195 genes that positively associated with* STIM1* score were identified (Bonferroni-adjusted* p*-value < 0.05). 21 significantly enriched Reactome pathways (false discovery rate (FDR) < 0.1; Supplementary Figure S13) were further identified 36. As expected, we found that candidate genes were enriched in the “calnexin/calreticulin cycle” pathway (FDR = 2.14×10^-3^) and several immune-related pathways, including the “antigen presentation: folding, assembly and peptide loading of class I MHC” pathway (FDR = 0.067). Furthermore, enrichment in COPI system or XBP1, IRE1-α, insulin receptor and mTORC1 signaling was also identified. These results thus provide an independent line of evidence that *STIM1*-mediated pathway enrichment may not necessarily tie with SOCE signaling, because *STIM1* score is able to capture SOCE-independent biological functions.

### Orchestration of *STIM1*-mediated cell-to-cell interactions in melanoma through ligand-receptor binding and cell infiltration

In contrast to immune cell types, the correlation between* STIM1* expression and immune-related pathways in malignant cells remains undefined. Given that the communications between malignant cells and immune cells by secreting cytokines/chemokines were widely observed, we delineated potential* STIM1*-mediated malignant-to-immune cell interactions by identifying cytokines/chemokines that were positively associated with* STIM1* expression in malignant cells. A curated list of ligand-receptor interactions 41 was adopted to pinpoint receptor gene(s) that correspond to* STIM1*-coexpressed cytokines/chemokines (*i.e.*, ligands secreted from malignant melanoma cells). In total of 9 ligands (including *TNFSF13*, *CCL5*, *IL16*, *IL1B*, *CCL18*, *IL6*, *CCL3*, *CXCL2* and *CXCL12*) as malignant-cell-derived SCGs were identified (Supplementary Figure S14). By leveraging two additional melanoma cell line (*i.e.* malignant cell) expression datasets (GSE7929 [*n* = 11] and GSE1845 [*n* = 5]), the positive correlation (*i.e.*, Pearson's product-moment correlation coefficient > 0) of *STIM1* to 3 (of 9) ligands was successfully validated including *CXCL12*, *IL16* and *CCL5* (Supplementary Figure S15).

We next leveraged ligand-receptor binding information to identify plausible *STIM1*-mediated crosstalk between malignant cells and immune cells in TME. We imply that immune crosstalk in melanoma tissues is presented when immune cells in TME possess receptors complemented with ligands secreted from *STIM1*-mediated cytokine genes. The expression of receptor genes corresponding to 3 SCGs (ligands) in non-malignant cell types were further taken into consideration. Representative examples of ligand-receptor interactions were shown in Figure 3B.* CXCR4*, a chemokine receptor, was expressed in malignant cells, while its ligand (*CXCL12*) was expressed in T cells (including CD4Treg, CD4Th, and CD8T) and NK cells. Another *STIM1*-associated ligand-receptor pairs were further demonstrated by *IL16*-*CCR5* and* CCL5*-*CXCR3* (Figure 3B).

**Figure 4:**
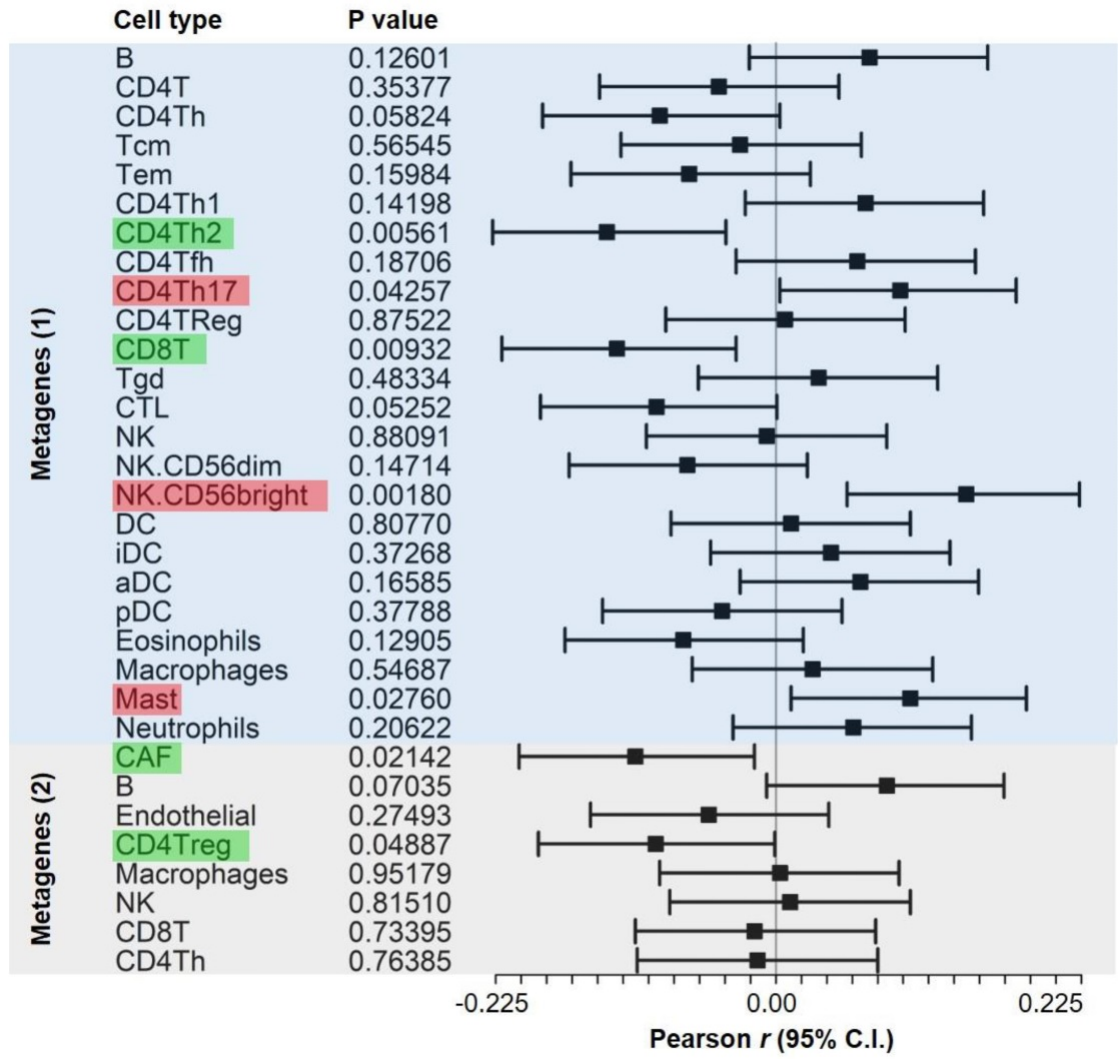
** Forest plot showing the statistics of correlations between cell-type metagenes and malignant-derived* STIM1* scores in bulk melanoma data.** Pearson's product-moment correlation coefficients between malignant-derived* STIM1* scores from specimens of The Cancer Genome Atlas (TCGA) skin cutaneous melanoma (SKCM) dataset and cell type metagenes (as well as their 95% confidence intervals (CIs)) are shown. Significant metagenes (with* p* < 0.05) with positive (Pearson's* r* > 0) and negative (Pearson's* r* < 0) associations are colored red and green, respectively. Genes that were used to construct cell type metagenes (2) were respectively adopted from (1) Bindea* et al.* 38 and (2) our previous study 37.

Genes that were specifically expressed in diverse immune cell types have been used as gene signatures to construct metagenes for quantifying the abundance of infiltrated cells in bulk tumor specimens 42. Accordingly, we investigated potential* STIM1*-mediated cell-cell interactions by associating malignant-derived* STIM1* scores with metagenes from 277 bulk TCGA melanoma transcriptomes with tumor purity (IHC) > 80%. Cell type-specific genes for metagenes calculation were adopted from previous work 38 (denoted as “Metagenes (1)”) and ours 43 (denoted as “metagenes (2)”). We quantified the correlations between malignant-cell-derived* STIM1* score and metagenes using Pearson's product-moment correlation coefficient (Figure 4). A significant positive association was observed between the malignant-cell-derived* STIM1* score and CD4^+^ T-helper 17 (CD4Th17) cell, CD56^+^ NK (NK.CD56bright) and mast cell. Additionally, negative associations were found between the malignant-cell-derived* STIM1* score and CD4^+^ T-helper 2 (CD4Th2) cell, CD8^+^ T (CD8T) cell, CAF and CD4^+^ Tregs (CD4Treg). Although association analysis can't determine causality, the results at least indicated a functional role of *STIM1*-mediated modulation in cell-cell interactions within the tumor microenvironment (TME).

### SCGs derived from immune cells indicate patient prognosis in melanoma

While previous studies have revealed the associations between immune infiltration and the prognosis of melanoma patients 15, 44-46, specific cellular programs that predict survival have not been reported. We tested whether* STIM1*-mediated cellular programs are associated with overall survival (OS) of melanoma patients. Correlation of clinical outcomes from the TCGA melanoma dataset with malignant-cell-derived* STIM1* scores were conducted. To prevent confounding from cell types with potential high *STIM1* score, we further restricted the bulk sample with tumor purity of > 80% (based on immunohistochemistry (IHC)), resulting in 277 (of 471, 58.8%) patients. After adjusting for gender and age at diagnosis, we detected no significant association between malignant-cell-derived* STIM1* scores and stage of cancer, depth of invasion, lymph node metastasis, distant metastasis, or OS (all* p*-values > 0.05; Supplementary Figure S16). These findings were further supported by 4 independent melanoma expression datasets (GSE22155 [survival endpoint: OS], GSE65904 [survival endpoint: disease specific survival (DSS)], GSE19234 [survival endpoint: OS] and GSE53118 [survival endpoint: OS]; Cox-proportional hazard model *p*-values > 0.05; Supplementary Figure S17).

**Figure 5:**
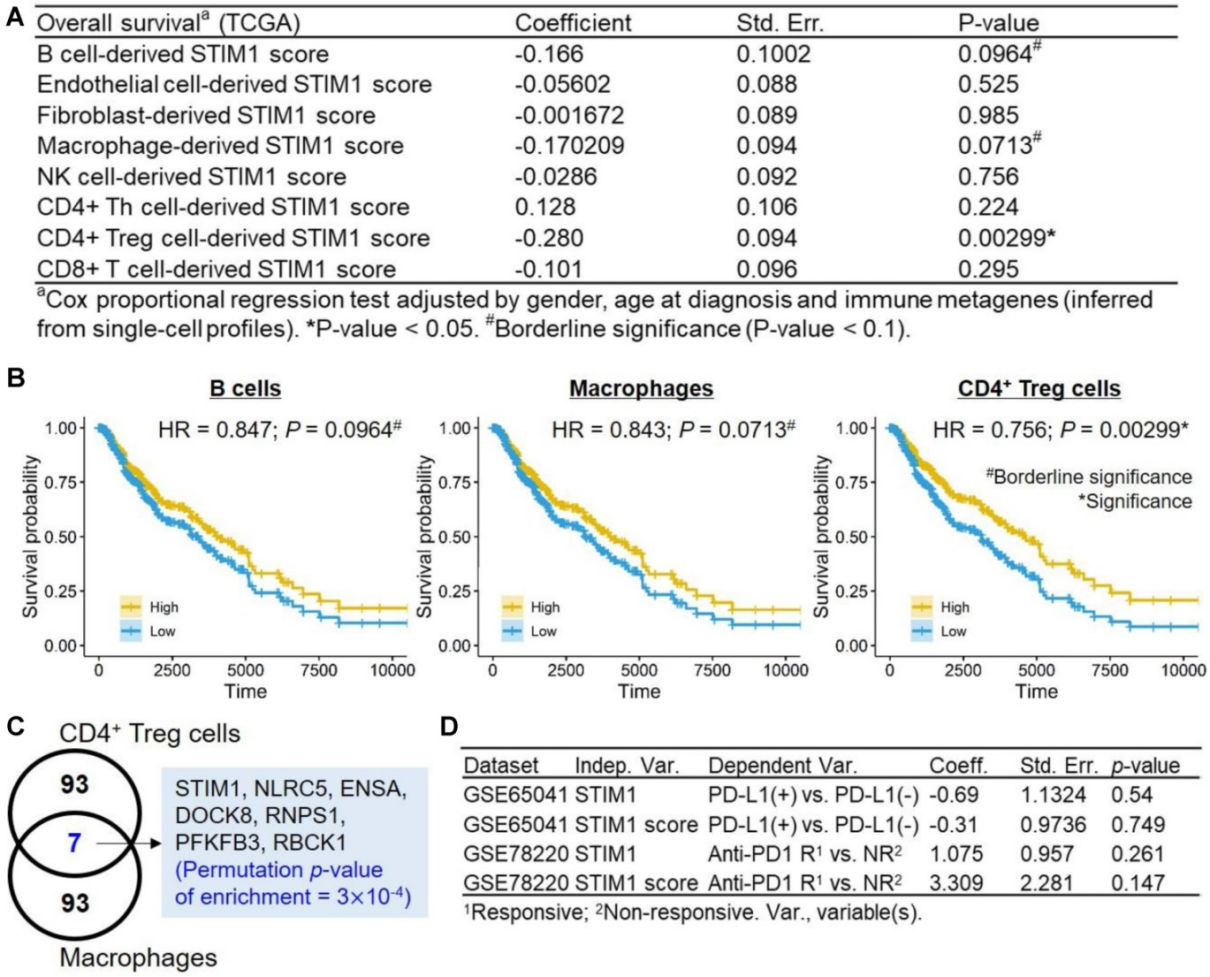
** Survival analysis of* STIM1* scores derived from non-malignant cell types.** (**A**) Clinical correlations of non-malignant cell-derived *STIM1* scores. (**B**) Kaplan-Meier plots (*x*-axis: time in day) showing survival curves of The Cancer Genome Atlas (TCGA) skin cutaneous melanoma (SKCM) patients with high (above the median) and low (below the median)* STIM1* scores derived from B cells (left), macrophages (middle), and CD4^+^ T regulatory cells (right). Hazard ratios and corresponding* p* values were calculated using the Cox-proportional hazard model by adjusting for gender, age at diagnosis, and immune metagenes. (**C**) Venn diagram showing the number of intersections between the top 100 SCGs derived from CD4^+^ T regulatory cells and macrophages. (**D**) Correlation (logistic regression test) between *STIM1* gene and *STIM1* score and the PD-L1 status (positive or negative; adjusted by gender and tumor status [primary or metastatic]) and responsiveness to anti-PD1 therapy (responsive [partial response and complete response] and non-responsive [progressive disease]; adjusted by gender and age) in melanoma patients.

We conducted similar regression tests for* STIM1* scores derived from diverse non-malignant cell types, while using TCGA samples without filtering for tumor purity (sample no. = 471; Figure 5A). A prognosis*-*favorable association (*p*-value = 0.00299, hazard ratio (HR) = 0.756) were identified in CD4^+^ Treg-derived* STIM1* score. Moreover, borderline prognosis*-*favorable associations were identified in B cell-derived* STIM1* score (HR = 0.85; *p*-value = 0.0964) and the macrophage-derived* STIM1* score (HR = 0.843; *p*-value = 0.0713; Figure 5B). We noted that the significance of CD4^+^ Treg-derived* STIM1* score should be interpreted with caution since both CD4^+^ Treg cells and macrophages exhibit high basal* STIM1* scores. Finally, a low specificity of CD4^+^ Treg-derived *STIM1* score were observed, which is suggested by significant enrichment of overlapping genes between the top 100 CD4^+^ Treg-derived SCGs and macrophage-derived SCGs (permutation* p*-value = 3×10^-4^; Figure 5C). Therefore, we observed the presence of *STIM1*-asociated clinical impact in immune cells (especially CD4^+^ Tregs) which is absence in malignant cells, suggesting functional versatility of *STIM1* in TME.

Immunotherapeutic implications of TME immune cell types had been characterized 47, given the prognostic significance of *STIM1* score in immune cell types based on the above results, it is valuable to ask whether *STIM1* correlates to immune checkpoint therapeutic outcomes. Regretfully, we detected neither significant association between *STIM1* gene expression (and *STIM1* score) to the PD-L1 expression status (positive *vs.* negative; GSE65041; *n* = 11) nor responsiveness to anti-PD1 (responsive [complete or partial response] *vs.* non-responsive [progressive disease]; GSE78220; *n* = 27) therapy in melanoma patients (all logistic regression test *p* > 0.05; Figure 5D).

## Discussion

Our analyses offer a single-cell-based transcriptomic metric that can be used to dissect multiple pathophysiological roles of* STIM1* expression in diverse cell types from melanoma-derived samples, while at the same time, providing an integrated view of* STIM1*-mediated alterations in cellular programs as well as prognostic correlations across different cell types in tumors. Utilizing a bottom-up approach starting with* STIM1* expression status, we identified genes that are co-expressed (positively and significantly associated) with* STIM1* in diverse cell types in melanoma and constructed* STIM1* scores to explore the biological relevance (Figure 1A). Further integration of SCGs from diverse cell types to the expression of bulky tumors links the functions of* STIM1* to patient prognosis and illustrates the cellular ecosystem in melanoma. In light of the fact that *STIM1* plays a critical role in immunity and has been linked with many cancers, our findings depicted its potential mechanisms in human melanomas.

The results from current study imply that* STIM1* expression in melanoma affects cellular physiology in a cell-specific manner. Specifically, malignant-cell-derived SCGs exhibit pathway convergence, whereas SCGs in non-malignant cell types do not (Figure 3). Therefore, malignant cell types may be a good starting point to investigate the functional versatility of* STIM1* expression in melanoma. In this study, we utilize* STIM1* score as a surrogate of* STIM1* expression in order to gain four advantages: 1) reduction in false negatives; 2) addition of information regarding* STIM1*-mediated cellular programs, which may not be reflected by* STIM1* expression alone (Supplementary Figure S12); 3) production of superior scoring in human melanoma tissues when compared to cell lines 17; and 4) delineation of malignant* STIM1* expression in bulky melanoma data by diminishing confounding effects from non-malignant cell types 5 (Figures 1C and E). Notably, the fourth advantage cannot be extended to non-malignant cell types because of the low specificity of* STIM1* scores derived from these cell types (Figure 2). Furthermore, we cannot distinguish whether the SCGs were located in the up- or down-stream of *STIM1*-mediated signaling. Here, we also simply ruled out the possibility of some SGCs as *STIM1*-unrelated genes and identified by coincidence in association, which is one of the pitfalls of this study. Given the result in Figure 1C, we can't simply rule out the contributions of some immune cell types (CAFs, macrophages and CD4^+^ Treg cells) to the final *STIM1* score, especially use these SCGs for bulk melanoma tissues. One way to correct the bias is filtering for tumor purity (*e.g.*, > 80% as in this study).

We thus provide a method to dissect malignant-cell-specific* STIM1*-dependent cellular programs in bulky melanoma tissues, which is difficult to accomplish without single-cell level data. Integration of malignant-cell-derived SCGs with data from bulk melanoma specimens and Reactome pathways further connects* STIM1* to alterations in calcium signaling, cell cycle regulation, and unexpectedly, immune-related cellular pathways in malignant melanoma cells (Figure 3A and Supplementary Figure S13). Since the store-operated Ca^2+^ entry (SOCE) pathway has been shown to be important in calcium signaling in non-excitable cells (such as malignant cells), our results couple* STIM1* expression with aberrant Ca^2+^ signaling, an idea which is supported by previous studies that indicate* STIM1* overexpression modulates SOCE in melanomas 11, 48-50 and other cancer types 9, 10, 51. In the present study, we focused on *STIM1* (not including *ORAI1*) only. One possible drawback is *STIM1* gene alone may not fully capture the calcium-dependent cellular process (especially SOCE), with previous study had suggested the role of Orai1-to-STIM1 ratio in determining the activation of CRAC current (I*_CRAC_*) 52. Therefore, the SOCE amplitude will be determined by relative expression between *ORAI1* and *STIM1* (and also other *STIM1* isoforms and *ORAI* isoforms) rather than the absolute level of *STIM1* alone. Here, since the major aim of this study is to characterize the *STIM1*-associated cellular programs in TME of melanoma, we selected to deliberate on SCGs (and *STIM1* score), which may capture both Ca^2+^-dependent and Ca^2+^-independent pathways across diverse cell types. Hence, these SCGs not only implicate in calcium signaling, but also may implicate in Ca^2+^-independent pathway(s) as illustrated in Supplementary Figure S13. Regarding SOCE amplitude, previous studies had revealed several gain-of-function mutations in *STIM1* (*e.g.*, D76A and E87A 53, L74P 54 and R304W 55), which may lead to constitutively Ca^2+^ influx and thus increase the basal Ca^2+^ level. Therefore, whether more gain-of-function mutations of *STIM1* in melanoma tumors is worth for investigation in the future.

In this study, we make the first connection between malignant* STIM1* expression and immune regulation in melanoma tissues. An illustrative example was shown in Figure 3B. The co-expression of* STIM1* with* CXCL12* (*a.k.a. SDF1*) was detected in malignant cells. CXCL12 may bind to the chemokine receptor, CXCR4, on CD8^+^ T cells, CD4^+^ T regulatory cells, CD4^+^ T helper cells, NK cells and macrophages, triggering downstream immune signaling pathways 56. Our analysis further links* STIM1* score to the abundance of immune cells in melanoma tissues (Figure 4), providing another mechanistic link between* STIM1* and immune-related pathways. Although the causality between malignant-cell-derived* STIM1* score and immune abundance cannot be absolutely delineated, our study, at least provided important clues for further investigations of* STIM1*-mediated malignant-immune cell interactions. Moreover, we gave an integrated view of how malignant-cell-expressed* STIM1* exerts its biological functions and mediates downstream cellular programs in diverse non-malignant cell types in the tumor microenvironment.

In addition to demonstrating the biological and clinical features of malignant-cell-derived* STIM1* score, this study also revealed* STIM1*-associated cellular programs in non-malignant cell types. Construction of a* STIM1* score (derived from non-malignant cells) in bulky melanoma tissues with available clinical data allowed us to examine the correlation between patient survival and* STIM1*-mediated cellular programs from non-malignant cell types (Figure 5A). For example, a significant favorable prognostic association of CD4^+^ T regulatory cell-derived* STIM1* score, as well as two borderline prognostic associations with B cell and macrophage-derived* STIM1* scores were established. In contrast, we failed to identify the association between malignant-cell-derived* STIM1* score and patient's prognosis. That may due to the diverse cellular functions elicited by* STIM1*.

Cancer cellular ecosystem is defined by an intricate network of cellular interactions that elicit downstream physiological interactions. By using single-cell transcriptomic profiles to define the role of* STIM1* expression in specific cells, we provide a systems-level view of* STIM1*-mediated cellular programs in TME of melanoma. Since calcium signaling is crucial for communication between malignant and stromal cells, maintaining growth and expansion in the cancer stroma 57, our study highlights the importance of integrated signaling between diverse cell types, including malignant cells, CAFs, endothelial cells, and immune cells, by revealing* STIM1*-associated cellular and clinicopathological features. In summary, this study advances the understanding of cell-specific* STIM1*-dependent biological alterations in human melanoma.

## Supplementary Material

Supplementary figures and table.

## Figures and Tables

**Table 1:** Validating the pipeline by comparing genes that were positively (*MITF*-co-expressed genes) and negatively associated with* MITF* to genes implicated in the* MITF*- and* AXL*-programs.

	Gene	Rank^a^	Beta coeff.^b^	Std. err.^c^	*Z*-value	*p* value	FDR^d^
Top ranked genes to the* MITF*-program	*MITF*	1	0.366	0.005	76.984	< 10^-20^	< 10^-20^
	*TYR*	2	0.126	0.014	9.204	3.45×10^-20^	8.50×10^-19^
	*PMEL*	3	0.058	0.009	6.495	8.29×10^-11^	4.30×10^-10^
	*PLP1*	4	0.095	0.010	9.053	1.39×10^-19^	3.08×10^-18^
	*GPR143*	5	0.097	0.009	10.332	5.03×10^-25^	2.70×10^-23^
Top ranked genes to the* AXL*-program	*ANGPTL4*	1	-0.077	0.011	-6.894	5.43×10^-12^	3.46×10^-11^
	*FSTL3*	2	-0.046	0.012	-4.027	5.66×10^-5^	1.08×10^-4^

^a^The rank of genes in each cellular program (*AXL*-program or* MITF*-program) was adopted from Tirosh* et al*. ^b^Beta coefficient of the association test. ^c^Standard error. ^d^ False discovery rate.

**Table 2:** Enrichment results of* STIM1*-coexpressed genes (SCGs) in a ranked gene list calculated from laser-captured micro-dissected melanoma tissues (*n* = 11).

First* N*_max_ partition	Top 25	Top 50	Top 100	Top 150	Top 200
20%	0.009277*	0.02065*	0.03515*	0.001123*	1.827×10^-5^*
30%	0.01066*	0.02368*	0.04029*	0.001299*	2.141×10^-5^*
40%	0.01194*	0.02631*	0.04459*	0.001449*	2.411×10^-5^*

*P < 0.05.* N*_max_, the number of genes considered in deriving the statistic of mHG test.

## Abbreviations

*STIM1*: stromal interaction molecule 1
SCGs: *STIM1*-coexpressed genes
ER: endoplasmic reticulum
CRAC: Ca^2+^ release-activated channel
CAF: cancer-associated fibroblast
LCM: laser capture microdissection
HGNC: Human Gene Nomenclature Committee
*t*-SNE: *t*-distributed stochastic neighbor embedding
EDM: exponential dispersion model
FDR: false discovery rate
MTC: multiple testing correction
ORA: over-representation analysis
TCGA: The Cancer Genome Atlas
GTEx: Genotype-Tissue Expression
NK: natural killer
OS: overall survival
HR: hazard ratio
SOCE: store-operated Ca^2+^ entry
