# Supplementary material for: Single-cell melanoma transcriptomes depicting functional versatility and clinical implications of STIM1 in the tumor microenvironment
Source: Theranostics. 2021 Mar 5;11(11):5092–106. doi: 10.7150/thno.54134 (PMC8039943; doi:10.7150/thno.54134)
Supplement: Supplementary file 1 — Supplementary figures and table. [file thnov11p5092s1.pdf]

SUPPLEMENTARY INFORMATION

Single-cell melanoma transcriptomes depicting functional versatility and clinical implications of STIM1 in the tumor microenvironment

	cy53	cy58	cy59	cy60	cy65	cy67	cy71	cy72	cy74	cy75	cy78	cy79	cy80	cy81	cy82	cy84	cy88	cy89	cy94	Sum
Malignant cell	16	0	**54**	9	4	0	**54**	0	0	0	**120**	**468**	**125**	**133**	32	14	**115**	**98**	10	1252
T cell	72	117	0	79	41	65	23	117	118	338	1	295	212	43	24	59	109	201	126	2040
B cell	0	2	0	95	5	19	0	35	13	0	1	78	49	3	1	25	16	106	64	512
Macrophage	12	2	1	4	1	0	2	0	5	1	0	0	0	0	4	21	38	26	2	119
Endothelial cell	10	0	0	0	0	0	0	0	0	0	0	2	27	2	0	1	0	1	19	62
NK cell	10	4	0	10	0	1	0	1	1	0	0	1	3	0	2	7	9	1	1	51
Fibroblast	4	0	7	0	0	0	0	0	0	0	1	1	20	0	5	1	2	0	15	56
Total	124	125	62	197	51	85	79	153	137	339	123	845	436	181	68	128	289	433	237	4092

Supplementary Figure S1. The cell number (in total 4092 cells) of each cell type from 19 metastatic melanoma patients. For malignant cells, 8 samples (cy59, cy71, cy78, cy79, cy80, cy81, cy88 and cy89) with cell number greater than 50 were separately included in the following study in considering the effect of inter-tumor heterogeneity. For non-malignant cell types, cells were included in the downstream analysis without filtering.

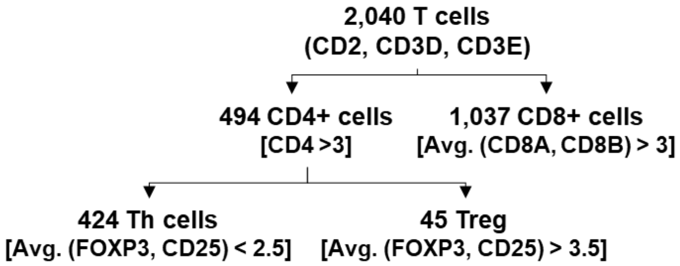

Supplementary Figure S2. 2040 T cells were further categorized into 4 T-cell subtypes (CD8+ T, CD4+ Th, CD4+ Treg, and other T) based on expression pattern of marker genes.

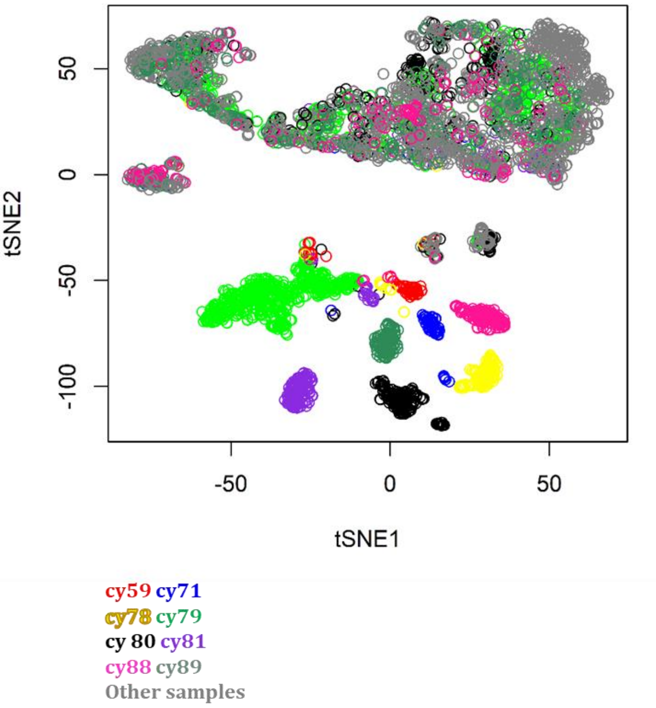

Supplementary Figure S3. The t-SNE clustering of single-cell transcriptomic profiles from 19 metastatic melanoma patients.

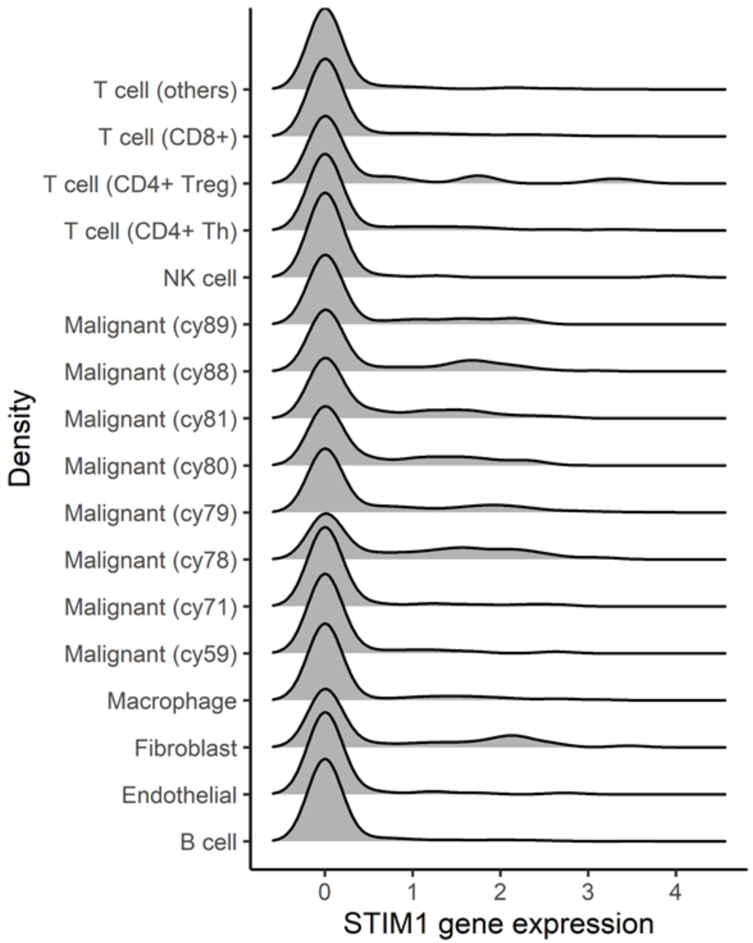

Min.	1st Qu.	Median	3rd Qu.	Max.	Mean
0	0	0	0	3.561581	0.134626
0	0	0	0	2.73688	0.136599
0	0	0	1.416945	3.456931	0.650084
0	0	0	0	3.138056	0.265122
0	0	0	0	2.633	0.167423
0	0	0	0	2.651275	0.195061
0	0	0.262468	1.575564	3.207182	0.803228
0	0	0	0.687589	3.692497	0.476774
0	0	0	1.0348	2.46878	0.479511
0	0	0	0.748803	2.783175	0.451316
0	0	0	0.954839	3.100848	0.509672
0	0	0	0.203209	2.318636	0.359758
0	0	0	0	3.969793	0.126345
0	0	0	0	3.602353	0.254464
0	0	0	0.303836	3.491503	0.454879
0	0	0	0	3.836224	0.222542
0	0	0	0	3.547636	0.15484

Supplementary Figure S4. Density plots showing the distributions (y-axis: density) of STIM1 gene expression (x-axis) across different cell types. The STIM1 gene expression values were represented by log_2(TPM/10+1) from single-cell sequencing (after quality controls and false-negativity correction).

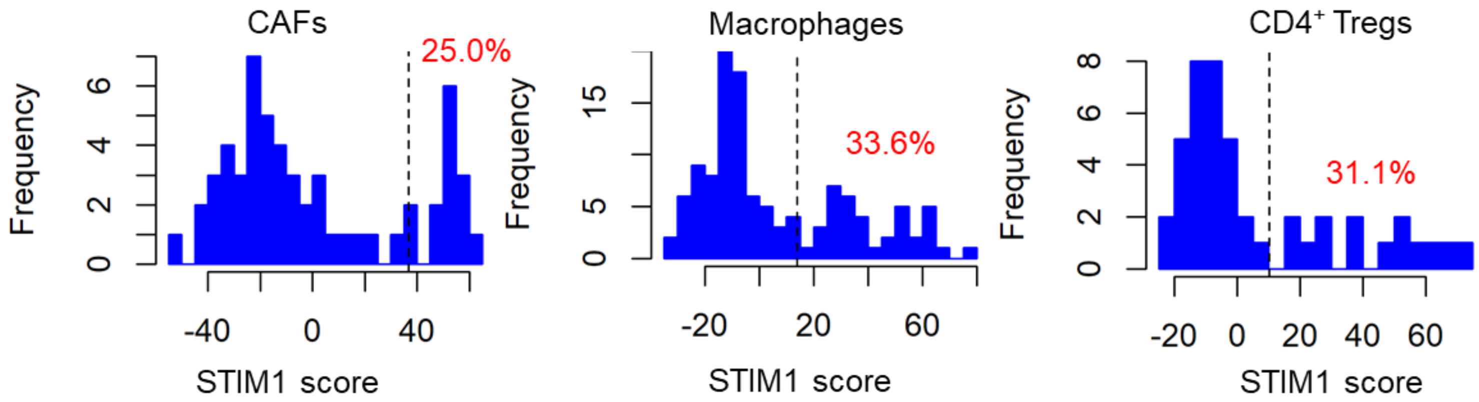

Supplementary Figure S5. Distribution plots of malignant-derived STIM1 score in CAFs, macrophages and CD4+ Tregs. The cutoff for each cell type was determined as follows: (1) first, STIM1 scores were fitted by using mixture model (bimodal normal distribution with cluster size of two); and (2) cutoff was then determined by Pr[drawn from low-STIM1 score cluster] =5%. For CAFs, macrophages and CD4+ Tregs, the cutoffs for STIM1 score level are 36.7, 14.0 and 10.1, respectively. The red value was the percentage of cells with STIM1 score greater than the cutoff in each cell type.

Type (**GSE4587**)	sample_size	STIM1	STIM1_score
atypical nevi	2	5.449164	7.578309
benign nevi	2	4.878978	7.312529
lymph node metastasis	3	5.530757	8.950681
melanoma in situ	2	5.698918	8.077382
metastatic melanoma culture	1	6.014578	9.574526
MGP melanoma	2	5.290497	8.7722
short term cultures of epidermal melanocytes	1	6.080018	9.265
VGP melanoma	2	5.023876	8.847176

- Melanoma (orange) vs. melanocyte (blue): p=0.5455
- Melanoma (orange) vs. nevus (green): p=0.008
- Nevus (green) vs. melanocyte (blue): p=0.4

Type (**GSE4570**)	sample_size	STIM1	STIM1_score
metastatic melanoma culture	5	8.015791	10.77978
normal melanocyte culture	2	8.208194	10.66728
primary melanoma culture	1	7.571373	10.83831

- Metastatic (orange) vs. melanocyte (blue): p=0.1905
- Metastatic (orange) vs. primary (yellow): p=1
- Metastatic (orange) + primary (yellow) vs. melanocyte (blue): p=0.1429

Type (**GSE3189**)	sample_size	STIM1	STIM1_score
Melanoma	45	8.272947	10.96047
Nevus	18	8.501918	10.61556

- Melanoma (orange) vs. nevus (green): p=0.001802

Supplementary Figure S6. Comparison of STIM1 gene expression and malignant-derived STIM1 score between melanoma, nevi, normal skin and melanocyte using 3 GEO datasets (GSE4587, GSE4570 and GSE3189). Mann-Whitney tests were performed for p-value calculation.

Comparison	Z	P.unadj	Type
B cell - T cell (CD8+)	3.323998679	0.000887366	intra-non-malignant
Malignant (cy80) - T cell (CD8+)	3.299985923	0.000966897	inter
Malignant (cy78) - T cell (CD8+)	3.255940828	0.001130173	inter
Fibroblast - Malignant (cy80)	-3.108858931	0.001878114	inter
Fibroblast - Malignant (cy78)	-3.098336082	0.001946106	inter
Malignant (cy79) - Malignant (cy80)	-2.838608059	0.004531077	intra-malignant
Malignant (cy78) - Malignant (cy79)	2.807803642	0.004988063	intra-malignant
B cell - Fibroblast	2.607527756	0.009119868	intra-non-malignant
B cell - Malignant (cy79)	2.390733942	0.016814733	inter
Fibroblast - Malignant (cy81)	-2.382702259	0.017186088	inter
Malignant (cy80) - NK cell	2.281678629	0.022508321	inter
Malignant (cy78) - NK cell	2.277055096	0.022782936	inter
Fibroblast - Malignant (cy89)	-2.171548038	0.02988977	inter
Malignant (cy80) - T cell (CD4+ Th)	2.151442065	0.031441324	inter
Malignant (cy80) - T cell (others)	2.134586217	0.032794824	inter
Macrophage - Malignant (cy80)	-2.133240812	0.032904977	inter
Malignant (cy78) - T cell (CD4+ Th)	2.132232684	0.032987723	inter
Macrophage - Malignant (cy78)	-2.123554785	0.03370739	inter
Malignant (cy78) - T cell (others)	2.114375123	0.034483241	inter
Malignant (cy81) - T cell (CD8+)	2.08562376	0.037012717	inter
Fibroblast - T cell (others)	-2.049042036	0.040458005	intra-non-malignant
Fibroblast - T cell (CD4+ Th)	-1.975941943	0.048161353	intra-non-malignant

Supplementary Figure S7. Dunn test for multiple comparisons of malignant-derived STIM1 score across cell types. Only the pairs of p-value less than 0.05 were shown.

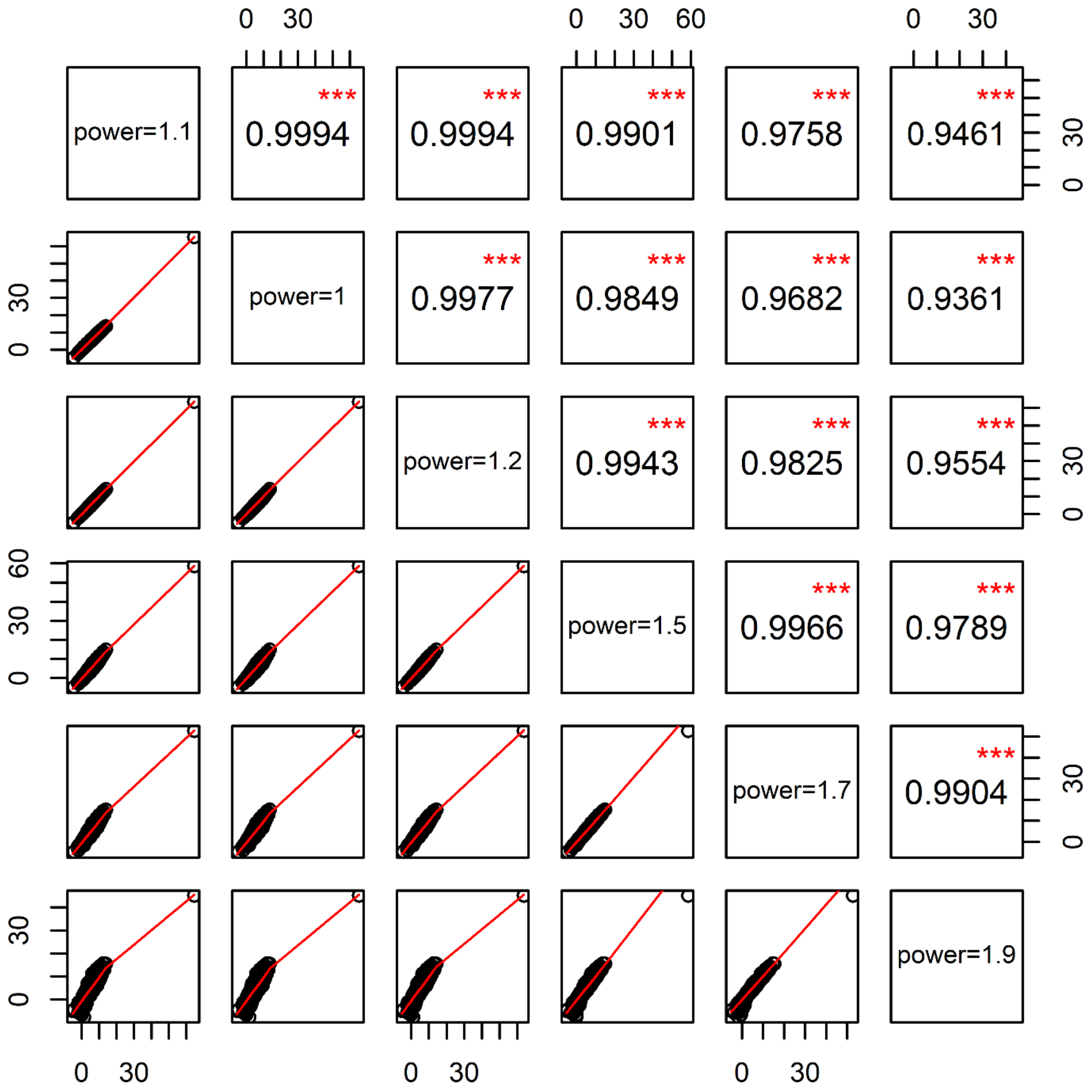

Supplementary Figure S8. Correlogram of Z-statistics from exponential dispersion models (EDMs) with different power parameter thresholds.

First N_{max} partitions	Top 25	Top 50	Top 100	Top 150	Top 200
20%	0.007823*	0.01807*	0.03961*	0.06104	0.08229
30%	0.009011*	0.02075*	0.04497*	0.06858	0.09191
40%	0.01011*	0.02292*	0.04934*	0.07485	0.09984
**P*-value less than 0.05.					

Supplementary Figure S9. Enrichment results of STIM1-associated genes in ranked gene lists calculated from GTEx skin tissues (n=974).

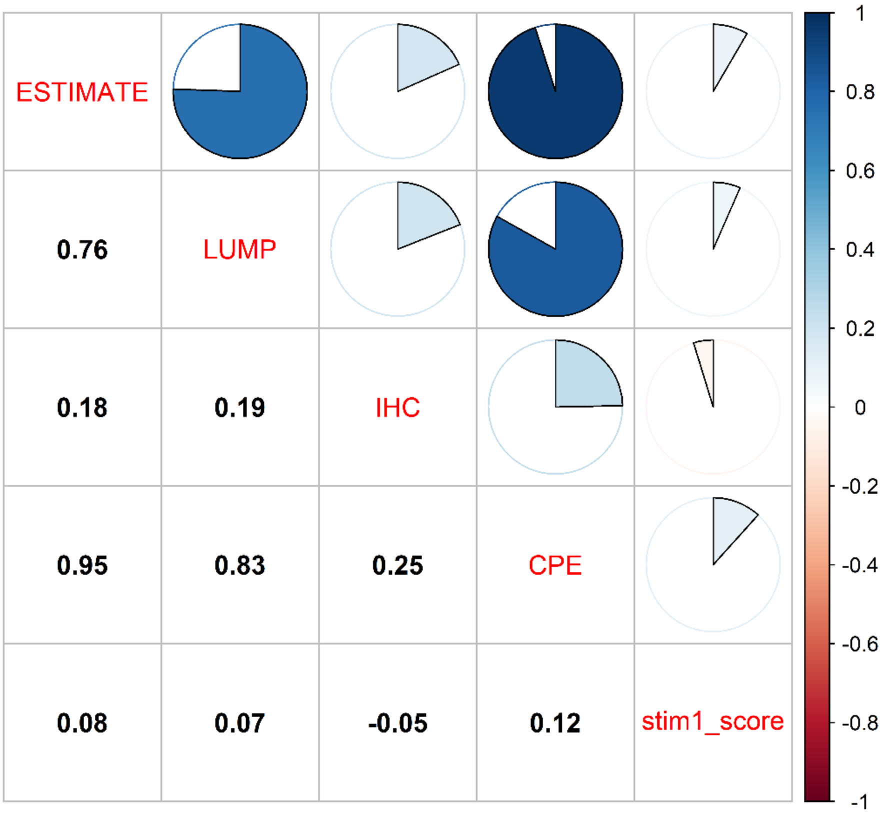

Supplementary Figure S10. Pearson's product-moment correlation coefficients between malignant-derived STIM1 score and 4 tumor purity indices from TCGA samples.

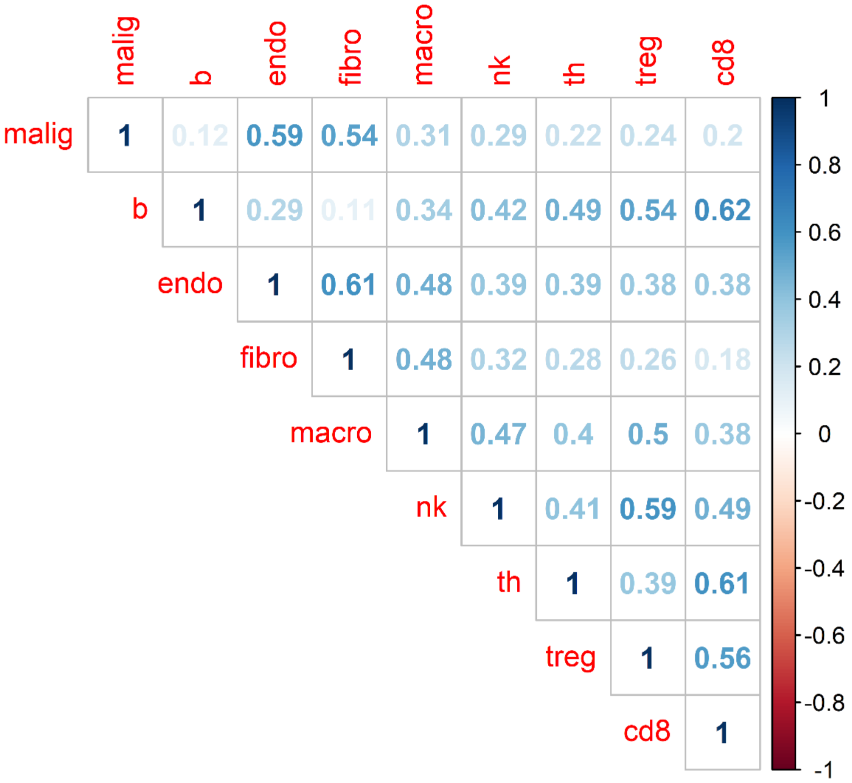

	malig	b	endo	fibro	macro	nk	th	treg	cd8
malig	1	0.12	0.59	0.54	0.31	0.29	0.22	0.24	0.2
b		1	0.29	0.11	0.34	0.42	0.49	0.54	0.62
endo			1	0.61	0.48	0.39	0.39	0.38	0.38
fibro				1	0.48	0.32	0.28	0.26	0.18
macro					1	0.47	0.4	0.5	0.38
nk						1	0.41	0.59	0.49
th							1	0.39	0.61
treg								1	0.56
cd8									1

Supplementary Figure S11. Pearson's product-moment correlation coefficients between STIM1 score derived from diverse malignant and non-malignant cell types.

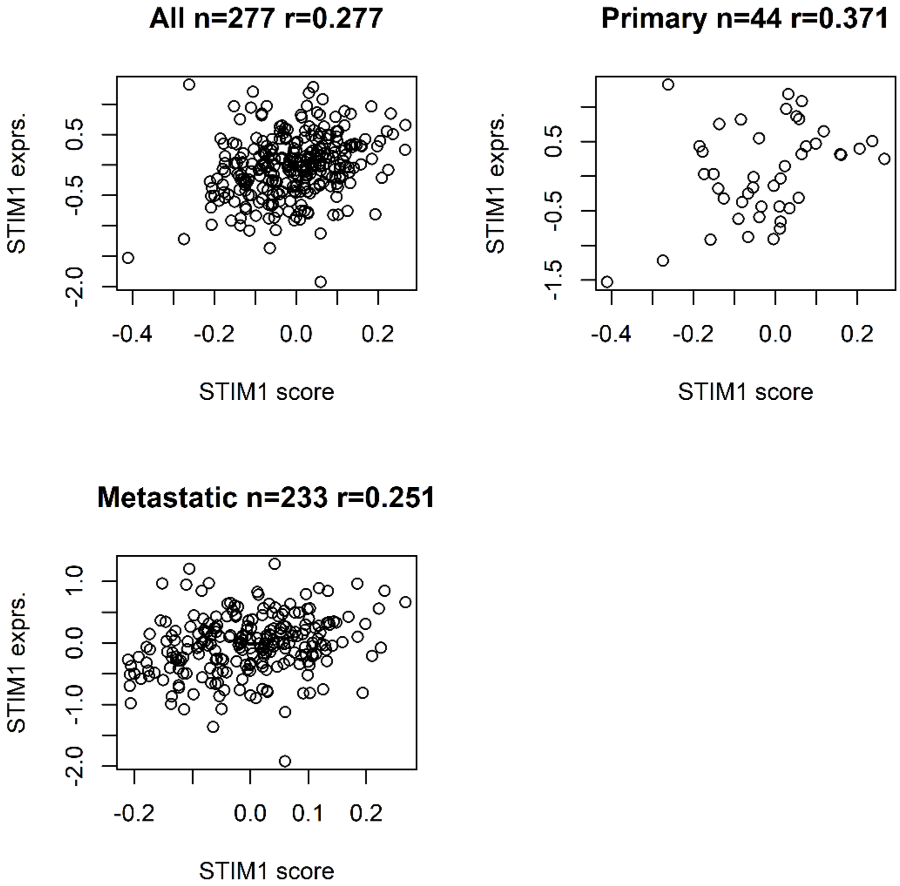

Supplementary Figure S12. Weak correlation (Pearson's r: 0.251 to 0.371) between STIM1 gene expression and malignant-derived STIM1 score calculated from top 100 malignant-derived STIM1-coexpressed genes from TCGA samples (IHC tumor purity >80%).

ID	Description	pvalue	p.adjust
R-HSA-446203	Asparagine N-linked glycosylation	2.12E-08	1.09E-05
R-HSA-6798695	Neutrophil degranulation	2.39E-07	6.17E-05
R-HSA-901042	Calnexin/calreticulin cycle	1.24E-05	0.002135
R-HSA-6807878	COPI-mediated anterograde transport	2.76E-05	0.003136
R-HSA-6811442	Intra-Golgi and retrograde Golgi-to-ER traffic	3.05E-05	0.003136
R-HSA-532668	N-glycan trimming in the ER and Calnexin/Calreticulin cycle	5.63E-05	0.00483
R-HSA-9612973	Autophagy	8.96E-05	0.006187
R-HSA-199977	ER to Golgi Anterograde Transport	9.61E-05	0.006187
R-HSA-6811434	COPI-dependent Golgi-to-ER retrograde traffic	0.000183	0.01002
R-HSA-8856688	Golgi-to-ER retrograde transport	0.000195	0.01002
R-HSA-77387	Insulin receptor recycling	0.000242	0.011335
R-HSA-948021	Transport to the Golgi and subsequent modification	0.000382	0.016385
R-HSA-917977	Transferrin endocytosis and recycling	0.000486	0.019216
R-HSA-432722	Golgi Associated Vesicle Biogenesis	0.000541	0.019216
R-HSA-162658	Golgi Cisternae Pericentriolar Stack Reorganization	0.00056	0.019216
R-HSA-5694530	Cargo concentration in the ER	0.000621	0.019301
R-HSA-917937	Iron uptake and transport	0.000637	0.019301
R-HSA-428157	Sphingolipid metabolism	0.000688	0.019688
R-HSA-1632852	Macroautophagy	0.000971	0.026312
R-HSA-204005	COPII-mediated vesicle transport	0.001317	0.033922
R-HSA-199992	trans-Golgi Network Vesicle Budding	0.001702	0.041741
R-HSA-1660662	Glycosphingolipid metabolism	0.002196	0.051405
R-HSA-74752	Signaling by Insulin receptor	0.002425	0.05431
R-HSA-381038	XBP1(S) activates chaperone genes	0.002987	0.064093
R-HSA-983170	Antigen Presentation: Folding, assembly and peptide loading of class I MHC	0.003211	0.066155
R-HSA-381070	IRE1alpha activates chaperones	0.003447	0.068278
R-HSA-9639288	Amino acids regulate mTORC1	0.004224	0.080574
R-HSA-381119	Unfolded Protein Response (UPR)	0.005418	0.099644

Supplementary Figure S13. Enrichment analysis for Reactome pathways of 195 STIM1-associated genes (Bonferroni-adjusted p-value <0.05) from TCGA data (n=277 with tumor purity >80% by IHC).

Ligand	Function	Beta	Std. Err.	Z-value	P-value	FDR[a]	Receptors
TNFSF13	Regulation of tumor cell growth	0.298	0.060	4.967	6.79×10^{-7}	1.88×10^{-6}	FAS, SDC2, TNFRSF11B, TNFRSF13B, TNFRSF14, TNFRSF1A
CCL5	Macrophage and NK cell migration	0.232	0.065	3.552	0.000382	0.000645	CCR1, CCR4, CCR5, CXCR3
IL16	Recruitment of CD4+ T cells	0.312	0.094	3.324	0.000887	0.001409	CCR5, CD4, KCNA3
IL1B	Inflammatory	0.245	0.085	2.875	0.004036	0.005718	ADRB2, IL1R2, IL1RAP
CCL18	Th2 response	0.138	0.053	2.605	0.009178	0.012224	n.a.
IL6	Inflammatory and costimulatory action	0.376	0.146	2.582	0.009827	0.013024	F3, IL6R, IL6ST
CCL3	Macrophage and NK cell migration	0.160	0.063	2.540	0.011091	0.014573	CCR1, CCR4, CCR5
CXCL2	Neutrophil trafficking	0.213	0.088	2.427	0.015229	0.01962	n.a.
CXCL12	Bone marrow homing	1.394	0.615	2.266	0.023435	0.02925	CCR4, CD4, CXCR3, CXCR4, ITGB1

[a]Cytokines/chemokines with beta coefficient > 0 and FDR < 0.05 were shown. n.a.: not available in single-cell data.

Supplementary Figure S14. Cytokines and chemokines (ligands) that positively associated with STIM1 gene (from single-cell malignant melanoma profiles). The statistics and significance values were calculated as previous described (methodology for identification of STIM1-coexpressed genes).

Dataset	TNFSF123	CCL5	IL16	IL1B	CCL18	IL6	CCL3	CXCL2	CXCL12
GSE7929	-0.42	0.66	0.24	0.52	0.22	0.40	n.a.	-0.23	0.49
GSE1845	-0.52	0.04	0.26	-0.76	-0.88	-0.47	n.a.	-0.86	0.67

Supplementary Figure S15. Validation of STIM1-associated ligands (cytokines/chemokines) using two additional datasets (GSE7929 and GSE1845). The number indicated the Pearson's product-moment correlation coefficient of expression level of ligand gene to the STIM1. The ligands genes showed positive correlation to STIM1 in both datasets were considered as successfully validated and highlighted in green color.

Clinical characteristics (TCGA samples with IHC >80%)	Coefficient	Std. Err.	P-value
Stage[a]	0.006505	0.008	0.4155
Depth of Invasion[a]	0.002131	0.0063	0.7366
Lymph Node Metastasis[a]	0.000707	0.0065	0.9133
Distant metastasis[a]	0.0127	0.0135	0.4945
Overall Survival[b]	0.030215	0.9958	0.9758

[a]Robust linear regression test adjusted by gender and age at diagnosis.
[b]Cox proportional regression test adjusted by gender, age at diagnosis.

Supplementary Figure S16. Clinical correlation of malignant cell-derived STIM1 score in 277 TCGA samples with tumor purity of >80% (by IHC).

dataset	type	coef	hr	s.e.	zval	pval
GSE22155	stim1	-0.169632522	0.843974902	0.281821732	-0.601914268	0.547231206
GSE22155	stim1_score	0.546101514	1.726509109	0.702102147	0.777809207	0.4366815
GSE65904	stim1	0.100581908	1.105814213	0.284950345	0.352980475	0.724103078
GSE65904	stim1_score	0.616525932	1.852481204	0.729960802	0.844601423	0.398333414
GSE19234	stim1	-0.309159672	0.734063551	0.613437232	-0.503979308	0.614275918
GSE19234	stim1_score	1.248092721	3.483692245	0.859886597	1.451462001	0.146651256
GSE53118	stim1	-0.073474843	0.92915952	0.235578483	-0.311891146	0.755123251
GSE53118	stim1_score	0.192247591	1.211970553	0.93374792	0.205888107	0.836878316

Supplementary Figure S17. Survival analysis of STIM1 gene or malignant-derived STIM1 score in melanoma patients. The hazard ratio (hr), standard error (s.e.) and p-values (pval) were estimated and calculated using Cox regression test.

Supplementary Table 1

rank	B cell			Endothelial			Fibroblast			Macrophage			NK cell			T cell (CD4+ Th)			T cell (CD4+ Treg)			T cell (CD8+)			T cell (others)			Malignant		
	gene	beta	sig	gene	beta	sig	gene	beta	sig	gene	beta	sig	gene	beta	sig	gene	beta	sig	gene	beta	sig	gene	beta	sig	gene	beta	sig	gene	beta	sig
1	STIM1	1.71	146.76	STIM1	1.78	17.16	STIM1	1.23	20.02	STIM1	1.59	37.73	PSMD8	1.81	15.15	STIM1	1.38	124.86	STIM1	1.13	14.08	STIM1	1.45	311.84	STIM1	1.61	148.87	STIM1	1.40	350.00
2	HLA-DRB6	1.18	33.49	POLR2B	1.71	10.79	DPYSL2	0.74	8.61	WBP2	0.95	8.83	FRG1	1.99	13.40	HNRNPR	0.72	24.28	NSFL1C	1.31	6.46	INPP4B	0.73	38.95	ARL6IP1	0.74	34.09	CD59	0.52	42.95
3	INPP5D	1.11	27.89	IMMT	1.41	9.21	CTSA	1.13	8.57	SND1	0.95	7.73	PNRC2	1.29	13.09	ANP32B	0.62	23.54	ACTN4	1.23	6.08	FYN	0.71	37.84	INPP4B	1.26	33.56	GANAB	0.44	36.85
4	HLA-DPB1	0.90	26.92	SDHC	1.75	8.26	HEXA	1.01	8.50	GTF2F1	1.18	6.81	SATB1	1.43	12.72	ARL6IP5	0.60	22.35	OTUD5	0.77	6.04	NLRC5	0.87	37.40	TMC8	1.16	31.98	PRKAR1A	0.45	34.07
5	SNX29	1.36	19.59	RNF4	2.06	8.05	ECHS1	1.05	8.31	CTBS	0.80	6.57	SLC25A24	1.52	12.68	COX6C	0.71	21.62	OTUB1	1.14	5.70	CBLB	0.78	36.87	LCP2	1.13	31.70	CALU	0.41	33.51
6	FCRL1	0.94	19.37	RNF167	2.04	7.54	APRT	0.75	8.26	OS9	0.74	6.43	ATXN7L3B	1.08	11.84	MAP4K1	0.89	21.55	TFRC	0.91	5.70	LCP2	0.87	36.72	SUN2	1.17	31.34	UBA1	0.46	32.77
7	ATP6V1G1	1.00	19.27	RPAIN	1.39	7.54	RPL36A-HNRNPH2	0.72	8.00	DOCK8	1.26	6.25	GUK1	1.90	11.83	XIST	0.90	21.47	C11orf54	8.47	5.43	ITGA4	0.77	36.00	RAB8B	1.30	28.88	CANX	0.42	32.61
8	ADAM19	1.29	18.91	RBM10	1.33	7.49	MDH1	0.81	7.94	CCT4	0.83	6.05	STIM1	1.25	11.65	FLII	0.78	21.05	NLRC5	1.40	5.33	ZNF207	0.70	33.93	COX6C	0.71	28.55	CNP	0.46	32.29
9	STK17A	1.22	17.84	USB1	1.28	7.37	SRSF4	0.86	7.94	GID8	1.12	5.64	RAB8A	1.02	11.43	LEPROTL1	0.89	20.99	ENSA	1.30	4.93	TAP1	0.57	33.81	NLRC5	1.06	26.67	ST13	0.44	31.44
10	ST6GAL1	0.91	16.15	LAMB2	1.34	7.36	CTTN	0.89	7.91	PPT1	0.84	5.43	RAP1B	1.04	11.19	SEPT6	0.82	20.60	C21orf91	1.16	4.90	EVL	1.00	33.48	SEC31A	1.02	26.15	YWHAE	0.41	31.10
11	TMC8	1.29	16.05	SEC61A2	1.53	7.02	EIF4H	0.74	7.88	PARN	0.99	5.38	RTN3	1.45	11.03	TAP2	1.04	20.37	IARS	1.15	4.86	MAP4K1	0.79	33.00	UBA1	0.97	26.12	TOMM20	0.44	30.62
12	LINC00685	1.21	16.03	VMP1	1.15	6.75	CCNL2	0.86	7.84	RNPS1	0.75	5.36	CHTF8	1.23	10.84	G3BP1	0.72	20.27	LASP1	0.98	4.76	GPRIN3	0.77	31.97	CBLB	0.97	26.06	HSPA9	0.43	30.57
13	YPEL5	0.65	15.45	DPP8	1.32	6.68	UQCR10	0.77	7.82	ARIH2	1.20	5.34	BCL6	1.87	10.71	DHX15	0.71	20.24	LARP7	1.82	4.70	CALCOCO1	0.73	31.64	TMEM123	0.94	25.89	TRAM1	0.46	30.56
14	GIT2	1.19	15.31	LRRFIP2	1.65	6.63	WDR83OS	1.06	7.74	PDE4B	0.74	5.31	ATF7	4.47	10.47	UBE2D3	0.57	19.66	TMIGD2	4.34	4.65	BANF1	0.74	31.57	LASP1	0.87	25.88	SND1	0.46	30.49
15	SLC44A2	0.81	15.28	UBE2J1	1.96	6.59	MAPRE1	0.81	7.71	PLK3	0.57	5.28	EDF1	2.01	10.34	DR1	0.85	19.43	UBQLN1	1.94	4.53	CDC42SE1	0.81	31.57	RNF4	0.93	25.55	WBP2	0.47	30.34
16	UBE2N	0.93	15.16	NSFL1C	2.24	6.50	NSA2	0.83	7.57	JUN	0.53	5.25	FLOT1	1.68	10.28	EIF4H	0.56	19.37	C18orf25	1.23	4.44	TAP2	0.96	31.19	SEMA4D	1.20	25.50	TPP1	0.40	30.03
17	SEL1L3	0.80	14.78	CD164	1.13	6.50	TMEM50A	0.77	7.21	CDC42EP3	1.23	5.18	WDFY2	7.56	10.25	VMP1	0.61	19.34	SORL1	1.42	4.36	CDK5RAP3	0.82	30.62	SNX5	1.02	24.72	RAB7A	0.40	29.41
18	DOCK2	0.95	14.70	PRMT2	1.65	6.48	PIP5K1A	0.74	7.17	VCP	0.74	5.15	MRPL3	1.46	10.24	CNOT2	1.15	19.17	ATP1B1	0.75	4.20	GNG2	0.78	30.57	GNAS	0.63	24.59	VPS35	0.45	28.93
19	USP11	1.21	14.56	AP1M1	1.05	6.32	GORASP2	0.79	7.10	NCOA4	0.57	5.07	TSPAN3	1.64	10.18	PHTF2	0.92	19.16	KRT8	0.72	4.18	LASP1	0.81	30.44	SLC38A1	1.08	24.53	CCDC47	0.48	28.89
20	MEF2C	0.85	14.37	NEDD9	1.49	6.30	GABARAP	0.55	6.92	MTHFS	0.91	5.07	PRKACB	1.75	10.08	RBCK1	0.75	19.11	SLC44A2	0.75	4.15	PRKACB	0.75	30.38	FYN	0.90	24.42	NBR1	0.49	28.86
21	LRCH1	1.43	14.11	CCDC25	2.22	6.19	CGGBP1	1.00	6.89	ANP32B	0.70	5.06	TTC19	1.74	10.01	SRP72	0.93	18.97	BCL2L1	0.71	4.13	GANAB	0.82	30.00	LEPROTL1	0.86	24.38	ZNF706	0.51	28.64
22	IL4R	1.01	14.03	SLC41A1	1.10	6.18	SEC31A	0.69	6.87	NLRC5	1.07	5.04	MROH1	1.30	9.98	NUDT21	0.93	18.95	DOCK8	1.74	4.10	RNF19A	0.74	29.98	SP110	0.90	24.35	RPS27L	0.41	28.60
23	PTK2	1.12	13.99	TBCD	1.40	5.95	OS9	0.92	6.87	UBE2E2	0.60	5.01	SNRPD3	1.57	9.97	TYK2	0.75	18.88	CSK	0.63	4.04	PROSER1	0.94	29.79	VCP	0.83	24.21	IVNS1ABP	0.40	28.56
24	UBXN1	0.72	13.98	STT3A	1.13	5.91	ABCF3	0.82	6.78	NAAA	0.74	4.99	SMNDC1	2.19	9.93	ARHGEF1	1.16	18.71	ZC3H12A	0.47	4.01	AP1G2	0.71	29.54	DRAP1	1.04	24.08	SHC1	0.46	28.40
25	MICAL1	1.39	13.87	TSN	1.07	5.90	RTN3	0.68	6.75	PIGT	0.57	4.95	ARPC1B	1.26	9.86	ANXA6	0.53	18.70	WASH7P	3.02	4.01	ESYT1	0.70	29.40	LITAF	0.78	23.62	CAPRIN1	0.46	28.08
26	ITGA4	1.28	13.73	RAD23A	1.56	5.89	CIB1	0.70	6.74	CSNK1A1	0.82	4.94	RPRD1B	1.60	9.74	SYNCRIP	0.94	18.61	EXOC1	1.07	4.01	PILRB	0.83	29.34	AP1G2	0.99	23.54	NFE2L2	0.46	27.88
27	TSPAN3	0.74	13.67	RABGAP1	1.83	5.86	CDK4	0.63	6.68	LITAF	0.49	4.87	BSG	1.19	9.69	GPSM3	1.00	18.48	ANKRD13C	2.37	4.00	AUP1	0.67	29.31	ORMDL1	1.04	23.33	SORT1	0.50	27.48
28	YTHDF2	0.83	13.65	MAP1LC3B	1.11	5.82	SERPINB6	0.69	6.60	SEC24B	0.86	4.86	SLU7	6.62	9.61	PRR13	0.56	18.34	CHST11	1.80	3.98	PLCG1	1.02	28.86	SNURF	0.80	23.12	VMP1	0.41	27.41
29	MBD4	0.95	13.58	TOB1	2.02	5.79	GET4	0.75	6.58	TOMM20	0.98	4.86	CRKL	3.91	9.61	CYFIP2	1.02	18.27	ARHGAP30	0.65	3.89	LITAF	0.59	28.80	SLFN5	0.87	22.84	DDB1	0.44	27.41
30	MAP4K1	1.02	13.30	SSB	1.35	5.76	RAB1A	0.63	6.57	ADAM10	0.81	4.83	MGRN1	1.67	9.61	MBTPS1	0.93	18.25	NR4A2	1.18	3.87	SEMA4D	0.75	28.79	SEPT6	1.01	22.73	HSPD1	0.39	27.38
31	ARL6IP4	0.81	13.26	USP37	2.78	5.75	ATG13	1.27	6.54	ARFRP1	0.70	4.77	POLR2A	3.37	9.61	HIF1A	0.77	18.24	EFTUD2	0.85	3.84	VPS52	0.81	28.45	GYPC	0.89	22.68	CD151	0.47	27.25
32	SHOC2	1.16	13.19	LASP1	1.20	5.74	MVP	0.66	6.52	DLD	0.90	4.77	SF1	4.36	9.61	MCL1	0.92	18.23	STAT6	2.35	3.83	ARHGAP4	0.70	28.44	TRAPPC1	1.07	22.63	CBX3	0.42	26.98
33	INTS1	1.63	13.12	GALNT2	2.56	5.69	LAMB1	0.70	6.51	PRKRIP1	0.79	4.73	UHMK1	1.72	9.61	ITPKB	1.02	18.20	LY6E	0.66	3.80	CDC42SE2	0.75	27.86	TAX1BP1	1.10	22.38	CS	0.48	26.91
34	IRF2	0.80	12.91	UBAP2L	1.75	5.67	MOB1A	0.64	6.48	REEP5	0.83	4.72	DPY19L1P1	13.81	9.61	CD164	0.55	18.20	DDX60	1.06	3.80	PCNP	0.68	27.84	CNOT7	0.98	22.31	EXOC7	0.53	26.75
35	ZCCHC7	1.01	12.90	SRSF4	1.52	5.67	NDUFB11	0.83	6.40	EIF1B	0.68	4.72	ARHGDIA	2.33	9.61	TMC8	0.91	18.15	SF3B5	0.54	3.77	ZC3H7A	0.72	27.67	ARHGAP4	0.90	22.29	UBAP2L	0.46	26.54
36	HLA-DQB2	0.88	12.86	SMIM7	1.27	5.66	CNOT2	1.02	6.40	PHB2	0.63	4.71	PPP1R2	2.14	9.61	UBL5	0.59	18.15	YY1AP1	1.05	3.72	GPSM3	1.19	27.41	CCDC88C	1.38	22.04	SEC61A1	0.41	26.53
37	TMEM123	0.90	12.63	SEC14L1	1.35	5.65	CALD1	0.44	6.32	HNMT	0.74	4.70	ZNF253	6.51	9.60	UQCR10	0.66	18.06	RNPS1	0.73	3.69	TXLNA	0.71	27.40	EVL	1.05	22.01	RAB6A	0.48	26.45
38	TRIM22	0.81	12.56	CNP	1.38	5.58	MLF2	0.71	6.32	TRA2B	2.37	4.66	DAG1	12.68	9.60	LMF2	0.89	17.86	GNG2	0.98	3.69	CYLD	0.88	27.30	KCNAB2	1.17	21.99	SPCS2	0.44	26.42

39	PAIP2	0.58	12.51	UBQLN2	1.64	5.56	TBCA	0.77	6.28	RASGRP2	0.69	4.57	SYK	1.70	9.60	PARP1	0.79	17.83	GMIP	0.89	3.69	STARD7	0.67	26.80	PCNP	0.90	21.89	ATP6V1G1	0.41	26.35
40	SIGLEC14	1.19	12.47	ITFG1	1.02	5.49	CMTM6	0.76	6.26	PPP4C	0.52	4.56	XRN1	3.20	9.60	CSNK1A1	0.82	17.80	IL18	4.66	3.68	NUP210	0.89	26.71	FLII	0.97	21.88	ANKRD36BP1	0.43	26.34
41	PHTF2	1.13	12.40	UBE2H	3.56	5.48	HMGN1	0.72	6.26	DNAJC5	0.90	4.45	MEF2D	1.95	9.60	CD28	0.83	17.77	PNRC1	1.27	3.67	VMP1	0.61	26.67	EID1	0.76	21.86	PKM	0.31	26.27
42	STX7	1.13	12.37	SDHA	1.68	5.46	SPG7	0.86	6.24	USP19	0.90	4.42	FBXW8	12.56	9.60	NR4A2	0.92	17.75	FAM53B	1.23	3.67	ORMDL1	0.75	26.46	PSMF1	1.06	21.86	RBM4	0.40	26.03
43	GPSM3	1.10	12.33	REEP5	1.60	5.42	FN1	0.43	6.24	POLE3	0.66	4.40	RBM14	2.66	9.60	TCEA1	1.02	17.63	PFKFB3	1.36	3.65	MOB1A	0.57	26.46	MAT2B	0.76	21.85	AK2	0.43	25.78
44	TRPS1	1.90	12.29	CD2BP2	1.53	5.42	AP2M1	0.65	6.20	NAMPT	0.46	4.38	AP3D1	1.91	9.60	EID1	0.72	17.50	RNF6	0.73	3.64	STK17A	0.71	26.40	EMB	0.81	21.84	PTPN1	0.52	25.77
45	MARCH7	0.97	12.27	TRIB2	1.43	5.28	TXN	0.46	6.19	RHBDF2	0.68	4.37	RAB11A	10.74	9.59	PA2G4	0.80	17.50	RBCK1	0.72	3.64	ANXA6	0.55	26.21	CNST	1.11	21.68	RAB1A	0.44	25.71
46	KDM2B	1.82	12.25	ITGB1	1.16	5.27	AP3S1	0.83	6.15	ZFP36	0.50	4.35	ZNF426	8.24	9.59	KCNAB2	0.91	17.42	TNPO3	1.56	3.51	DR1	0.72	26.13	ARHGAP9	0.83	21.53	TNPO1	0.56	25.63
47	TAF11	1.20	12.23	IGBP1	1.13	5.26	SNRNP27	0.74	6.14	CYTH1	0.70	4.35	NDRG3	4.38	9.59	CDV3	0.95	17.42	ABR	0.91	3.51	ACTR3	0.50	26.07	UBE2D3	0.66	21.51	ACTN4	0.44	25.59
48	LCOR	2.23	12.02	LAYN	1.00	5.25	PSMA7	0.51	6.13	FBXL5	0.68	4.31	SRCAP	5.60	9.59	TXLNA	0.99	17.41	MYO18A	2.01	3.47	SRP19	0.80	26.05	ST13	0.99	21.43	DUSP4	0.45	25.58
49	ARL6IP1	0.78	11.95	DPP7	1.12	5.22	GIT2	1.13	6.12	GNB2	0.84	4.29	ERC1	23.31	9.58	CYTH1	0.72	17.41	ATG4B	0.93	3.44	FLII	0.67	25.90	STAT1	0.64	21.41	GDI2	0.39	25.31
50	AFTPH	1.21	11.80	PPM1F	1.22	5.21	ATF7	0.81	6.12	CCT5	0.73	4.24	ATF3	1.18	9.58	LASP1	0.87	17.35	NSUN5	0.92	3.43	ITPKB	0.88	25.76	MRPL10	0.93	21.27	RBMX	0.38	25.23
51	IKZF1	1.24	11.76	TMEM256	0.90	5.21	CTBS	1.20	6.11	TTC19	0.86	4.22	MRPL44	13.85	9.58	TPP1	0.83	17.32	DICER1	3.68	3.43	SMARCE1	0.71	25.70	SNRPD1	0.85	21.27	MAT2A	0.38	25.21
52	SEPT6	0.90	11.74	UBE2N	1.77	5.20	SDHD	0.79	6.10	CFD	0.50	4.21	FAM153C	4.71	9.58	DNAJB14	0.76	17.31	ZNF397	0.59	3.40	HGS	0.68	25.66	UBE2N	0.91	21.25	SEPT2	0.38	25.10
53	LRRC40	0.79	11.71	LPXN	1.17	5.18	DNAJC7	0.70	6.09	MAN2B1	0.61	4.21	TRAF5	4.72	9.58	RNF149	1.42	17.22	PDPK1	2.42	3.39	PTK2B	0.67	25.62	SNRPN	0.81	21.24	ZNF207	0.39	25.08
54	ESYT1	1.05	11.69	TNFRSF14	1.03	5.16	GPAA1	0.73	6.04	TMEM30A	0.74	4.19	TMEM50B	9.24	9.58	CDK17	1.06	17.21	PBXIP1	0.94	3.38	HIF1A	0.73	25.53	CDC42SE2	0.73	21.21	RALY	0.60	25.05
55	RB1	1.50	11.64	RNF40	2.25	5.13	AP2A1	0.85	6.02	GNAS	0.80	4.18	HNRNPA3	4.37	9.58	PAIP2	0.57	17.15	AFTPH	1.33	3.38	ARPC5	0.47	25.46	NFATC2	1.38	21.12	NPC1	0.42	24.98
56	AP1G2	0.85	11.58	SRPK1	2.13	5.12	RHEB	0.65	6.00	TUBGCP6	1.68	4.16	LAX1	4.03	9.57	SLFN5	0.73	17.15	APBA3	0.48	3.37	SLFN5	0.68	25.37	ACTR3	0.63	21.11	RER1	0.49	24.95
57	GAPT	0.86	11.56	CTBS	1.31	5.03	AEBP1	0.60	6.00	NEDD9	0.93	4.14	PDCL	8.29	9.57	FOXO1	0.89	17.11	MMADHC	0.48	3.36	TMC8	0.84	25.23	ERAP1	1.50	21.03	SQSTM1	0.39	24.94
58	CUX1	1.50	11.56	HSPD1	1.42	4.99	UAP1	0.55	5.99	ZNF692	0.59	4.14	FBXO48	17.14	9.57	POLR2J3	1.32	17.08	SEMA4D	1.87	3.35	SEC22B	0.80	25.14	ANP32B	0.74	20.91	ITGB1	0.41	24.83
59	SNX5	0.78	11.50	ATF6B	1.41	4.98	EID1	0.73	5.98	UGP2	0.68	4.13	VAPA	3.87	9.56	UBA1	0.82	16.84	LCOR	3.09	3.35	SAP30BP	0.79	25.07	R3HDM1	1.69	20.84	YWHAQ	0.44	24.76
60	CAPN7	1.54	11.36	CALCOCO1	0.98	4.98	MAP1B	0.57	5.97	ZFYVE26	1.25	4.08	ITPR2	2.22	9.56	RAD23A	0.92	16.82	PEA15	1.17	3.32	PPP2R5C	0.68	25.03	EIF4H	0.72	20.68	CD55	0.40	24.73
61	RNF114	0.83	11.33	PAGR1	1.68	4.96	LAMTOR5	0.82	5.95	CKAP4	0.84	4.08	LTN1	2.18	9.56	PSMF1	0.90	16.81	KAT5	0.52	3.32	CD46	0.64	24.96	CD2BP2	1.17	20.66	LSM12	0.53	24.73
62	FGD2	1.25	11.31	NDUFB11	1.58	4.96	KXD1	0.78	5.92	COMT	0.64	4.08	PAN3	2.65	9.56	TERF2IP	0.80	16.80	UBR1	2.17	3.30	RASA2	0.81	24.93	PHTF2	1.03	20.64	WBP11	0.50	24.72
63	SMIM7	0.70	11.30	RAC1	1.57	4.95	CDC42SE1	0.88	5.91	MAT2B	0.66	4.04	DCUN1D1	0.99	9.56	SPOCK2	0.72	16.71	CD164	0.58	3.30	CPNE1	0.59	24.92	ENSA	1.01	20.59	CSNK1A1	0.40	24.65
64	FBXL4	0.87	11.26	SEC61A1	1.31	4.93	HECTD1	1.16	5.91	PFKFB3	0.70	4.03	ZBED1	9.84	9.56	HIVEP2	1.05	16.66	FAM120A	3.83	3.29	HLA-DPB1	0.58	24.83	EIF5A	0.61	20.54	COX6C	0.40	24.59
65	RALGPS2	0.82	11.22	HLA-DRB6	1.30	4.93	WDR34	0.65	5.91	NUP93	0.61	4.03	ETS1	2.23	9.55	CDC42SE1	0.81	16.64	PSPC1	0.71	3.26	RAB1B	0.78	24.81	TMEM140	0.86	20.50	CALD1	0.49	24.55
66	CD55	1.20	11.15	ACTN4	1.69	4.91	MAN2C1	0.93	5.91	DPP7	0.57	4.03	PDE4DIP	2.10	9.55	ARHGAP4	0.69	16.63	RSBN1	7.24	3.25	HLA-DRB6	0.79	24.77	C18orf25	1.23	20.47	CNOT7	0.46	24.46
67	SPPL2B	1.04	11.14	PSMD6	0.86	4.91	ST13	0.72	5.90	ATAD2B	1.62	4.01	MCC	62.69	9.55	RBL2	1.21	16.58	HGS	0.75	3.24	CD2BP2	0.77	24.77	RER1	1.41	20.22	CDK16	0.45	24.42
68	RNF138	0.85	11.13	CCT6A	1.45	4.90	FAM160B2	0.72	5.89	CAPNS1	0.68	4.01	INGX	14.06	9.55	RNF167	1.01	16.56	FBLN1	4.65	3.24	RAVER1	0.90	24.75	ANXA6	0.69	20.17	AUP1	0.45	24.40
69	NSUN5	1.13	11.11	FLNB	1.38	4.90	GDI2	0.56	5.87	TBC1D10C	0.52	3.99	HERC1	4.45	9.54	RPL22L1	1.14	16.53	TTYH3	0.86	3.21	IL16	0.80	24.54	MAP4K1	1.06	20.13	CTNND1	0.48	24.32
70	C5orf15	0.77	11.10	CCL28	2.14	4.89	SMARCB1	0.86	5.86	MXD1	0.68	3.99	AKNA	4.30	9.53	MAT2B	0.64	16.53	MIER2	0.99	3.21	PRMT2	0.73	24.53	SPG7	1.17	20.07	GPBP1	0.61	24.31
71	DOCK8	1.41	11.06	ABLIM1	1.61	4.89	RCC2	0.89	5.86	TOM1	0.68	3.99	ANKRD10	2.10	9.53	UBE2N	0.72	16.52	INTS3	1.32	3.21	ST6GAL1	0.72	24.44	RBCK1	0.95	19.99	PSMD11	0.44	24.29
72	FARP2	1.21	11.02	MTUS1	0.99	4.87	MAP3K7	0.79	5.85	RBCK1	0.77	3.98	TNFRSF10B	21.15	9.52	FAM49B	0.82	16.48	SCAND1	0.46	3.20	KCNAB2	0.99	24.41	VPS39	1.06	19.92	TAX1BP1	0.42	24.26
73	SZRD1	1.92	10.95	RAB7A	1.15	4.86	RBM5	0.95	5.85	TRIM25	1.21	3.98	PRRC2A	3.86	9.51	ADAM10	0.85	16.47	GOLPH3	0.56	3.20	PRR13	0.59	24.35	TPM4	0.71	19.89	POLR2F	0.50	24.24
74	ZC3H7A	1.11	10.91	PEA15	1.55	4.82	ZNF770	1.36	5.84	TAZ	0.61	3.98	AP1G1	1.96	9.50	TMC6	0.69	16.46	GRINA	1.35	3.19	MCL1	0.71	24.24	ITPKB	1.25	19.89	MLPH	0.41	24.24
75	NR4A2	1.43	10.85	PLSCR1	1.51	4.79	CBX3	0.72	5.81	TNIP1	0.97	3.96	RBM8A	2.82	9.50	CANX	0.75	16.44	KIAA2026	29.31	3.19	CNOT7	0.76	24.20	NASP	0.94	19.75	PCBP1	0.38	24.21
76	SUN1	0.83	10.84	RFTN1	1.03	4.78	EPS15L1	0.93	5.80	RCSD1	0.57	3.94	ANKRD52	3.77	9.49	SEPT2	0.71	16.42	RNF167	1.04	3.15	RBCK1	0.66	24.17	TGFBR2	1.13	19.75	HSPE1	0.37	24.10
77	TSPYL2	1.00	10.84	PTGES3	1.43	4.76	DHX15	0.61	5.79	SDCCAG8	0.67	3.90	NOP56	1.39	9.49	DMTF1	1.13	16.41	PSEN1	1.41	3.15	RAP1B	0.50	24.15	PSMD3	0.98	19.60	STX7	0.44	24.08
78	BIRC3	0.97	10.76	EIF3C	1.40	4.70	HEXB	0.66	5.78	SULT1A3	0.74	3.90	NME6	11.11	9.49	PTEN	1.46	16.31	UBE2Z	0.84	3.15	STAG3L3	1.13	24.12	PPP1R2	0.99	19.60	OS9	0.43	24.08
79	DCAF6	1.54	10.73	SLC44A1	2.64	4.68	PTBP1	0.63	5.76	SULT1A4	0.74	3.90	RBM12	14.48	9.48	EVL	0.91	16.23	CLEC17A	5.52	3.15	ANP32B	0.58	24.04	UQCR10	0.82	19.58	TSPAN3	0.47	23.84
80	TMC6	0.83	10.73	ANP32B	1.38	4.67	ACTR3	0.43	5.75	ENSA	0.83	3.89	HIST2H2AA3	1.30	9.48	EIF3H	0.49	16.23	SFI1	1.44	3.14	DNAJC8	0.56	24.01	FAM102A	1.23	19.56	RAB1B	0.40	23.84

81	PRKCB	1.01	10.67	EDC3	1.06	4.59	LAS1L	0.69	5.73	LIMK2	0.63	3.88	HIST2H2AA4	1.30	9.48	PTGES3	0.56	16.18	ABCA2	1.73	3.13	SUPT5H	1.14	23.97	CLDND1	0.80	19.56	UBE2I	0.49	23.69
82	ATF6B	1.13	10.59	RNASEH2C	3.01	4.57	FXR1	1.27	5.73	TNFAIP2	0.53	3.88	PPP6R3	2.41	9.48	SLC38A1	0.92	16.18	ZC3H7A	1.00	3.13	LEPROTL1	0.68	23.94	ST6GAL1	0.98	19.49	ERBB3	0.36	23.66
83	CTNNBL1	0.60	10.56	SEPT2	1.52	4.56	TSN	0.76	5.72	CYBA	0.71	3.86	ITSN2	1.73	9.47	SMARCE1	0.70	16.16	SDHAP1	0.69	3.13	MAN2B2	0.95	23.94	OAS2	0.77	19.46	TIMP2	0.50	23.55
84	TANK	0.58	10.52	SH3BP5	0.95	4.55	EFCAB14	0.98	5.72	CAPN1	0.61	3.84	ROCK1	2.59	9.47	ATG4B	0.94	16.14	PES1	0.68	3.12	INPP5D	0.68	23.92	ZNF217	1.18	19.43	CD164	0.42	23.49
85	THADA	1.15	10.50	EML2	1.01	4.53	MED17	1.08	5.71	CHPF2	1.44	3.84	CHEK1	3.15	9.46	ATP6V1G1	0.61	16.10	SREBF2	1.87	3.11	SYVN1	1.58	23.88	PILRB	1.03	19.43	TMEM106B	0.64	23.48
86	DEPDC5	0.86	10.50	CRIPT	1.73	4.52	CYBRD1	0.67	5.70	FAM160A2	0.77	3.84	SRRM1	4.13	9.46	UBE2I	0.89	16.10	CEBPZ	1.12	3.11	PPM1G	0.55	23.80	MOV10	0.98	19.35	GDI1	0.45	23.41
87	AKAP2	0.80	10.47	NAMPT	1.04	4.51	PPP1R15A	0.62	5.69	TCF7	2.11	3.82	ACTR10	1.75	9.46	COTL1	0.55	16.09	TNS3	1.39	3.11	NFATC3	0.88	23.74	NOP56	0.81	19.32	PTGES3	0.38	23.40
88	SH3BP5	0.70	10.38	TSR3	1.18	4.49	EWSR1	0.72	5.68	LPAR6	0.57	3.81	CD3G	5.95	9.45	CSK	0.63	16.08	IMPA1	23.11	3.10	HSPH1	0.75	23.63	PNRC2	0.73	19.22	ATF6B	0.46	23.39
89	PSIP1	1.04	10.29	ADD1	1.92	4.47	EPRS	0.70	5.67	NAA10	0.50	3.81	HK2	35.02	9.45	YWHAE	0.73	16.07	CLIP1	1.24	3.10	TNIP1	0.96	23.62	RBMX	0.63	19.21	ATP1A1	0.44	23.35
90	IMMT	0.86	10.20	JMJD4	1.08	4.46	MRPS35	0.67	5.64	MCL1	0.77	3.81	ZNF461	10.79	9.45	PILRB	1.00	16.06	TMBIM1	0.63	3.08	XRCC5	0.61	23.60	IMMT	0.91	19.19	GNAS	0.39	23.21
91	NISCH	1.24	10.19	TJP1	1.61	4.45	TFE3	0.95	5.64	RAB1A	0.67	3.79	GPR65	2.86	9.45	FGFR1OP2	0.70	16.00	TAB2	1.08	3.07	ARRDC3	0.74	23.58	SHISA5	0.64	19.14	RAB5C	0.43	23.20
92	SLK	6.37	10.19	SPIN1	1.42	4.43	GBE1	0.57	5.64	PTP4A1	0.81	3.78	HNRNPD	1.93	9.45	RAB5C	1.05	16.00	ZNF26	7.78	3.06	NFATC2	1.08	23.49	TRERF1	1.16	19.11	UBE2D3	0.37	23.18
93	NBPF15	1.71	10.15	CHMP2B	1.62	4.43	THUMPD3	1.28	5.63	VPS4A	0.61	3.78	ARID1A	2.59	9.44	NLRC5	0.95	15.91	GPRIN3	0.93	3.06	ERICH1	0.87	23.43	POLR2M	0.95	19.11	NAMPT	0.40	23.18
94	SUN2	1.52	10.12	CLCN3	5.09	4.43	KDELR2	0.52	5.62	DGKD	0.93	3.77	CDK1	1.22	9.44	REEP5	0.78	15.88	RAB11B	1.35	3.06	TRPS1	0.80	23.10	AZIN1	1.11	19.08	DHX9	0.45	23.13
95	POU2F2	0.80	10.08	OS9	1.15	4.42	CEP63	1.45	5.62	PPM1F	0.99	3.77	ZFAS1	1.35	9.44	TMEM50A	0.74	15.86	UBA1	0.83	3.05	VCP	0.60	23.09	PKM	0.64	19.06	OTUB1	0.47	23.11
96	PNRC1	1.20	9.99	SCAMP3	0.92	4.41	CTDSP2	0.78	5.60	ALDH16A1	0.70	3.76	PI4KA	1.58	9.44	EHD1	0.71	15.84	GSE1	2.16	3.04	RBMX	0.57	23.06	MAPK8IP3	2.58	19.04	CAP1	0.35	23.07
97	ARRDC3	1.03	9.95	COX6C	1.20	4.40	PDIA3	0.56	5.60	APLP2	0.63	3.74	FLNA	2.45	9.43	LCP2	0.95	15.84	BCL9L	3.35	3.03	RBL2	0.78	23.04	SEC22B	0.99	19.00	PACSIN2	0.46	23.01
98	MSL3	0.98	9.94	MYO9A	1.93	4.40	CAPRIN1	0.68	5.59	DNAJA1	0.38	3.73	ARIH2OS	8.29	9.43	FYN	0.83	15.83	UBE4B	3.31	3.02	CHST11	1.08	23.02	GIT2	0.95	19.00	SH3KBP1	0.57	22.88
99	GAK	1.32	9.94	PRKAR1A	1.18	4.40	OSTF1	0.64	5.58	PAN2	2.28	3.73	CS	1.22	9.42	USP10	0.76	15.83	LCP2	1.36	3.02	SNX14	0.84	22.95	TBC1D14	0.93	18.96	TM9SF2	0.43	22.88
100	FAM49B	1.05	9.93	COPB1	1.37	4.38	MAN2B2	0.84	5.58	MAX	0.68	3.72	GNAS	1.30	9.41	SZRD1	0.99	15.81	DBNL	0.65	3.02	DDB1	0.74	22.95	ZNF207	0.72	18.89	TAF1D	0.51	22.82
Sig: -log10(P-value)																														
